# Role of *Saccharomyces cerevisiae* Nutrient Signaling Pathways During Winemaking: A Phenomics Approach

**DOI:** 10.3389/fbioe.2020.00853

**Published:** 2020-07-22

**Authors:** Beatriz Vallejo, Emilien Peltier, Victor Garrigós, Emilia Matallana, Philippe Marullo, Agustín Aranda

**Affiliations:** ^1^Institute for Integrative Systems Biology (I2SysBio), University of Valencia-CSIC, Valencia, Spain; ^2^Génétique Moléculaire, Génomique, Microbiologie (GMGM), University of Strasbourg, Strasbourg, France; ^3^ISVV UR Oenology, INRAE, Bordeaux INP, University of Bordeaux, Bordeaux, France; ^4^Biolaffort, Bordeaux, France

**Keywords:** *Saccharomyces cerevisiae*, wine, nutrient signaling, TORC1 pathway, PKA, Snf1 kinase, glucose repression, Gln3

## Abstract

The ability of the yeast *Saccharomyces cerevisiae* to adapt to the changing environment of industrial processes lies in the activation and coordination of many molecular pathways. The most relevant ones are nutrient signaling pathways because they control growth and stress response mechanisms as a result of nutrient availability or scarcity and, therefore, leave an ample margin to improve yeast biotechnological performance. A standardized grape juice fermentation assay allowed the analysis of mutants for different elements of many nutrient signaling pathways under different conditions (low/high nitrogen and different oxygenation levels) to allow genetic-environment interactions to be analyzed. The results indicate that the cAMP-dependent PKA pathway is the most relevant regardless of fermentation conditions, while mutations on TOR pathways display an effect that depends on nitrogen availability. The production of metabolites of interest, such as glycerol, acetic acid and pyruvate, is controlled in a coordinated manner by the contribution of several components of different pathways. Ras GTPase Ras2, a stimulator of cAMP production, is a key factor for achieving fermentation, and is also relevant for sensing nitrogen availability. Increasing cAMP concentrations by deleting an enzyme used for its degradation, phosphodiesterase Pde2, proved a good way to increase fermentation kinetics, and offered keys for biotechnological improvement. Surprisingly glucose repression protein kinase Snf1 and Nitrogen Catabolite Repression transcription factor Gln3 are relevant in fermentation, even in the absence of starvation. Gln3 proved essential for respiration in several genetic backgrounds, and its presence is required to achieve full glucose de-repression. Therefore, most pathways sense different types of nutrients and only their coordinated action can ensure successful wine fermentation.

## Introduction

*Saccharomyces cerevisiae* has been used as a very successful model organism to explain the molecular mechanisms regulating cell growth and metabolism (Conrad et al., 2014; Rodkaer and Faergeman, 2014; Zhang et al., 2018). A variety of molecular systems, called nutrient signaling pathways, sense the presence or absence of nutrients outside and inside the cell, and elicit a molecular response, generally at the transcriptional level, to regulate gene expression and to adapt metabolism to the changing environment. The sensing pathways of the two main nutrients (carbon and nitrogen) have been widely characterized under controlled laboratory conditions using laboratory strains. In the presence of glucose, which is a principal carbon source of laboratory media, cAMP-dependent protein kinase A (PKA) represses the stress response and stimulates fermentation and cell proliferation (Conrad et al., 2014). Adenylate cyclase produces cAMP when stimulated by two kinds of G-proteins: Ras1/2 and Gpr1. There are two Ras proteins in *S. cerevisiae*, Ras1 and Ras2, which sense intracellular glucose by mechanisms still to be completely defined. cAMP binds to the regulatory subunit of PKA, Bcy1, and then releases catalytic subunits and triggers the transcription response. The cAMP level is controlled by a negative feedback loop that involves phosphodiesterases Pde1/2, which regulates PKA activity. When sugar is low or absent, AMP-activated kinase Snf1 relieves glucose repression by enabling the use of alternative carbon sources. In nitrogen sensing, the main player is the TORC1 complex. TORC1 senses intracellular nitrogen availability, particularly the mobilization of amino acids from the vacuole through Gtr1 (Powis and De Virgilio, 2016). TORC1 catalytic kinases can be either Tor1 or Tor2. The complex has not only many targets, like protein kinase Sch9, which controls protein synthesis, but also many downstream branches that control different aspects of the metabolism and transport of amino acids, including the system called Nitrogen Catabolite Repression (NCR) (Zhang et al., 2018). NCR is activated by good nitrogen sources like ammonia and glutamine, and is repressed by poor sources like proline. This system contains two transcription factors, Gln3 and Gat1, and one repressor protein, Ure2. In addition to TORC1, and regarding amino acid signaling, the General Amino Acid Control kinase Gcn2 senses amino acid starvation to promote the biosynthesis of nitrogen compounds through transcription factor Gcn4 (Conrad et al., 2014).

Besides its contribution to understand the molecular architecture of cell signaling, the budding yeast *S. cerevisiae* is involved in the alcoholic fermentation of various raw materials, including worth, grape juice and bakery dough (Sicard and Legras, 2011). In the winemaking context, yeast transforms grape must hexoses into ethanol and CO_2_ by producing a large number of primary and secondary metabolites that play an important role in wine organoleptic properties (Ribéreau-Gayon et al., 2006). Growth conditions during winemaking are quite different from those of standard laboratory conditions. Indeed enological fermentations are characterized by a low oxygen level in the grape musts containing large amounts of fermentable hexoses and small quantities of nitrogen compounds and other micronutrients. In some cases, imbalances and deficiencies may lead to stuck or sluggish fermentations at a very high cost for industry (Bisson, 1999). For instance, yeast viability drops during lipid-limited fermentations when excess nitrogen is present (Tesniere et al., 2013). Carbon-to-nitrogen ratios are similarly relevant. Reducing TORC1 signaling by deleting *SCH9* extends chronological longevity in laboratory medium (with high nitrogen and low sugar), but shortens life spans under winemaking conditions (high sugar and low nitrogen) (Picazo et al., 2015). From the genetics point of view, wine strains constitute a well-defined group of the *S. cerevisiae* population (Peter et al., 2018). First, wine yeasts are mostly prototrophic and differ from the standard laboratory strains carrying various auxotrophic mutations used as selection markers. Second, some degree of genetic variation in nutrient signaling pathways, such as TORC1, has been found in natural isolates of *S. cerevisiae* (Kessi-Perez et al., 2019, 2020). Although they constitute a homogenous group, commercial wine starters show wide genetic variability, as reflected by their different sensitivity to inhibitors of nutrient signaling pathways (Vallejo et al., submitted).

**FIGURE 1:**
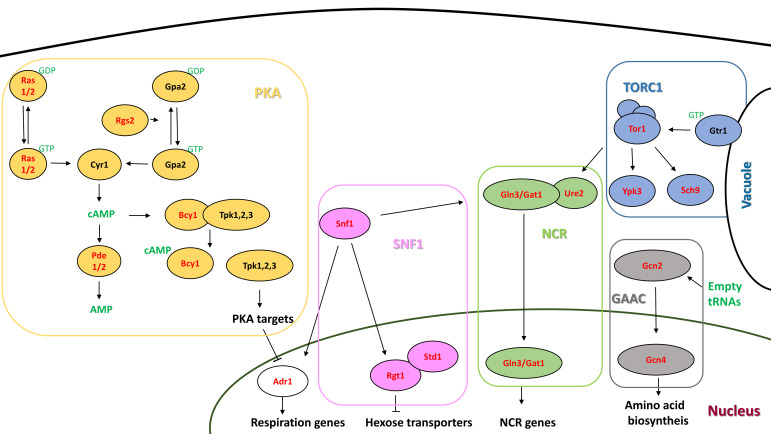
Schematic depiction of the main nutrient signaling pathways in *S. cerevisiae*. The proteins tested by deletion are indicated in red. Signaling molecules are indicated in green.

Genetic variability among wine starters and intrinsic variability in the composition of grape juices render the use of standardized fermentation methods necessary to assess quantitative enological traits (Peltier et al., 2018). In this work, such a fermentation device was used to test a variety of mutants on different nutrient signaling pathways (Figure 1) under three different enological conditions by changing nitrogen concentrations and oxygenation levels, which revealed interesting gene-environment (GxE) interactions and phenotypic connections between pathways. The results point out the main role of PKA in achieving good fermentation performance, while TORC1 is key for the production of primary metabolites, like glycerol. In addition, this phenotypic survey underlines some unexpected results, like the impact of AMPK Snf1 when grown under high sugar conditions, and its close relation to NCR transcription factor Gln3. Our results indicate that nutrient signaling pathway genetic manipulation can be a good target of performance improvement. It can help to increase metabolites of interests or fermentation speed, as shown by the activation of PKA by cAMP phosphodiesterase *PDE2* deletion.

## Materials and Methods

### Yeast Strains and Growth Conditions

The employed *S. cerevisiae* strains are described in Supplementary Table S1. Mutants were made in haploid wine strain C9 (Walker et al., 2003) with the reusable *kan*MX marker, which was amplified by PCR from the pUG6 plasmid (Guldener et al., 1996). This marker contains flanking *lox*P sites to excise it by employing the Cre recombinase from plasmid YEp-cre-cyh (Delneri et al., 2000). The CRISPR-Cas9 deletion of the *PDE2* gene was made using plasmid pRCC-K, which was a gift from Eckhard Boles (Addgene plasmid # 81191), in accordance with the provided protocol (Generoso et al., 2016). Yeast transformations were performed by the lithium acetate method (Gietz and Woods, 2002).

For standard propagation, yeasts were grown in rich YPD medium (1% yeast extract, 2% Bacto Peptone, 2% glucose). Solid plates contained 2% agar, and 20 μg/ml of geneticin for the selection of *kan*MX transformants. Minimal medium SD contained 0.17% yeast nitrogen base, 0.5% ammonium sulfate and 2% glucose (Adams et al., 1998). This medium was used to select the transformants with cycloheximide resistance by employing 2 μg/ml.

For the growth spot analysis, serial dilutions form stationary cultures in YPD were made and 5 μl drops were placed on selective media. The carbon source was changed in YPD using 2% glycerol or sucrose whenever necessary, as was the nitrogen source in SD by adding 0.5% proline instead of ammonium sulfate. 2-deoxyglucose was added at 200 μg/ml. Red grape juice (Bobal variety) was a gift from Bodegas Murviedro (Requena, Spain) and was sterilized overnight with 500 μg/l of dimethyl dicarbonate in cold. The microfermentations of the *PDE2* deletion mutant by CRISPR-Cas9 were made in conical tubes with 30 ml of juice at 24°C and gentle shaking (Orozco et al., 2012). CFU was followed by serial dilutions and plating on YPD plates. Sugars were measured with DNS (dinitro-3,5-salycilic acid) according to Miller’s method (Robyt and Whelan, 1972). Other metabolites were measured with commercial kits (Megazyme Ltd., Bray, Ireland).

### Grape Must for Standardized Fermentations

Fermentations were carried out in a Sauvignon Blanc grape must harvested in 2016 (SB16) in the Bordeaux Area, provided by Vignobles Ducourt (Ladaux, France). Before fermentation, grape must was sterilized by membrane filtration (cellulose acetate 0.45 μm Sartorius Stedim Biotech, Aubagne, France). Fermenting sugars (205 g/l) were estimated by measuring glucose and fructose following the enzymatic method described by Stitt et al. (1989). The Yeast Assimilable Nitrogen (YAN) content of SB16 was estimated enzymatically using enzymatic kits K-PANOPA and K-AMIAR (Megazyme) following the manufacturer’s instructions. SB16 contains 99 mg N/l of YAN (80 mg/N from primary amino acids and 19 mg N from ammonium), which represented the low nitrogen condition. A normal nitrogen modality containing 250 mg N/l was obtained by spiking SB16 with 170 mg/l of a mixture of amino acid and 80 mg/l of ammonium chloride. The composition of amino acid mixture is described elsewhere (Marullo et al., 2006).

### Fermentation Monitoring

Fermentations were carried out following the method described by Peltier et al. (2018). Briefly, 20 ml screwed vials (Fisher Scientific, Hampton, New Hampshire, United States ref: 11981523) were filled with 11.5 ml of grape must inoculated with 2 × 10^6^ viable cell⋅ml^–1^. The Screwed Vials (SV) were tightly closed with 18 mm screw cap magnetic −3 mm HT silicone/PTFE stoppers (Fisher Scientific, Hampton, New Hampshire, United States). Hypodermic needles (G26–0.45 × 13 mm, Terumo, Shibuya, Tokyo, Japan) were inserted into the septum for CO_2_ release. The fermentation temperature was maintained at 24°C by an incubator (Binder GmbH, Tuttlingen, Germany). When specified, vials were shaken at 175 rpm throughout fermentation using an orbital shaker (SSL1, Stuart, Vernon Hills, Illinois, United States). The fermentation kinetics was estimated by manually monitoring (2–3 times/day) the weight loss caused by CO_2_ release using a precision balance with automatic weight recording (Mettler Toledo, Greifensee, Switzerland). The amount of CO_2_ released according to time was modeled by the local polynomial regression fit to estimate the six kinetics parameters previously described (Peltier et al., 2018): the maximal amount of CO_2_ released (*CO_2_max* in g⋅l^–1^), the lag phase (lp in h), the time to release 35, 50% and 80% of maximal expected CO_2_ after subtracting lp (t35-lp, t50-lp and t80-lp in h) and the average hexose consumption rate between 50% and 80% of CO_2_max (V50_80 in g⋅l^–1^ ⋅h^–1^). The concentrations of the following organic metabolites were measured at the end of fermentation: acetic acid, glycerol, malic acid, pyruvate and total SO_2_ using the respective enzymatic kits: K-ACETGK, K-GCROLGK, K-LMAL-116A, K-PYRUV, and K-TSULPH, (Megazyme, Bray, Ireland) following the manufacturer’s instructions. Glucose and fructose were assayed by the enzymatic method described by Stitt et al. (1989).

### Alcohol Dehydrogenase Zymogram

An aliquot of a culture of each strain grown in YPD in the early exponential phase (OD_600_ 0.4–0.6) was collected and considered the glucose-repressed conditions. The culture was grown for 24 h more in the postdiauxic phase, which was taken as the derepressed conditions. Alcohol dehydrogenase was visibilized in non-denaturing PAGE (Dombek et al., 2004). Cells were broken in cold 50 mM phosphate buffer, pH 7.5, with glass beads in a FastPrep 24 (MP-Biomedicals) and run in cold 6.5% acrylamide PAGE gel (Williamson et al., 1980). Activity was detected in a mix of 2 mg phenasine methosulfonate (PMS), 5 mg nitrotetrazolium blue (NTB), 25 mg NAD and 0.05 ml ethanol dissolved in 25 ml of 0.1 M Tris–HCl buffer, pH 8.5 (Fowler et al., 1972).

## Results

### Validation of the Standard Fermentation Behavior of Haploid Wine Strain C9

The aim of this study is to make an overall assessment of the phenotypic impact of null mutations impacting the key genes involved in main nutrient signaling pathways. We measured this impact under different environmental conditions by following a previously validated standardized method. The used genetic background was a haploid derivative of wine commercial strain L2056 called C9, which is a convenient background for performing gene deletions (Walker et al., 2005). Since both ploidy and the strain background strongly influence wine fermentation phenotypes (Marullo et al., 2006), overall C9 performance was compared to other commercial starters routinely used in enology. A fermentation experiment was carried out with strains C9, EC1118, DV10, M2, CSM, and BQS252. EC1118 and DV10, which are related strains that belong to the *Prise de Mousse* clade (Borneman et al., 2016), while M2 and CSM are commercial strains used for red winemaking. According to the manufacturer, EC1118 and DV10 require low nitrogen, while M2 has high nitrogen requirements. CSM was defined in previous studies by our group as a strain with a long chronological life span, while EC1118 displays short longevity (Orozco et al., 2012). Therefore, these strains show a wide phenotypic variability with one another. Laboratory strain BQS252, from the S288c genetic background and prototrophic for amino acid synthesis, was used as a reference after previously proving its ability to finish fermentation (Vallejo et al., submitted). Three environmental conditions (NS:Low, NS:Normal, and S:Normal) were applied by changing the nitrogen content of SB16 (Low/Normal) as well as the fermentation shaking (Not Shaking/Shaking). Therefore, nitrogen differences were tested, while shaking is a condition to increase micro-oxygenation compared to non-shaking conditions (Peltier et al., 2018).

**FIGURE 2:**
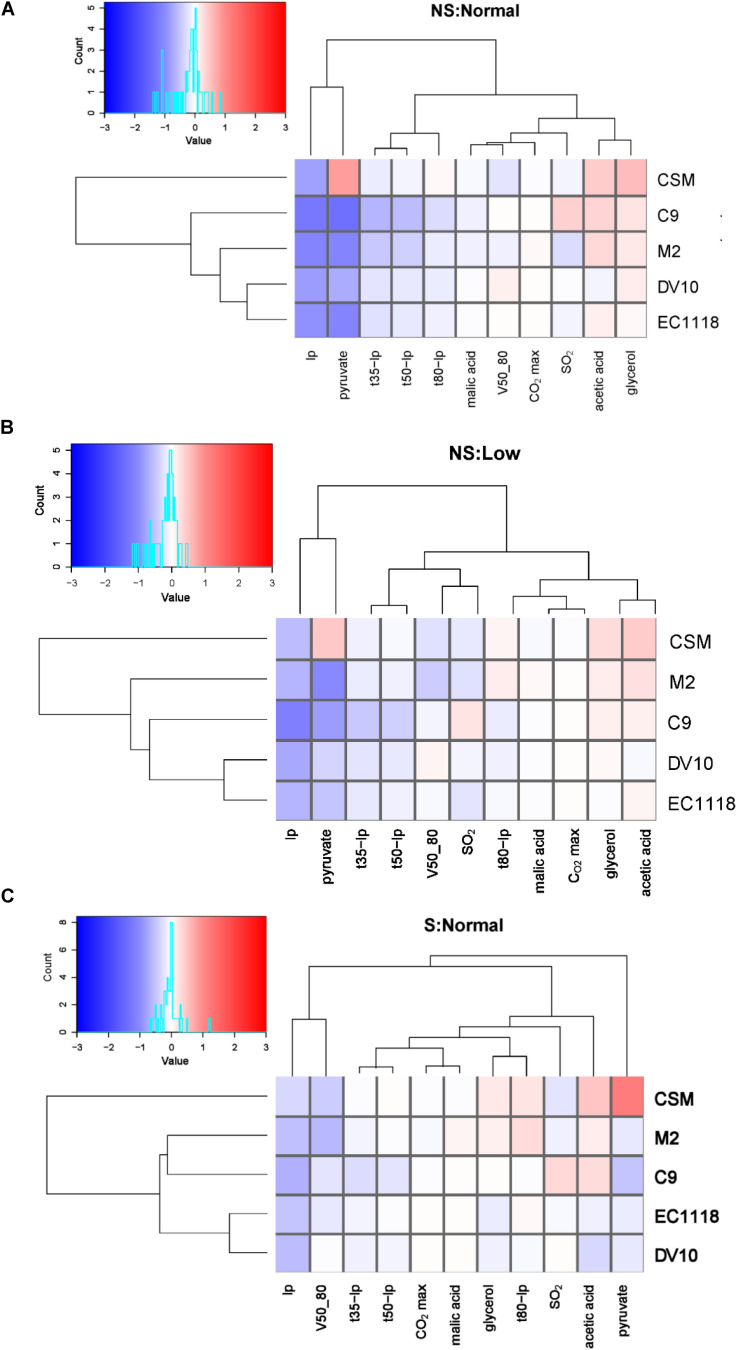
The haploid wine strain shows a similar behavior to commercial strains. Heatmaps showing relative changes in the kinetics and end-product concentrations of the selected wine strains (C9, CSM, EC1118, DV10, and M2) relative to the laboratory strain for three conditions. **(A)** No shaking: normal nitrogen; **(B)** No shaking: low nitrogen; **(C)** Shaking: normal nitrogen. Log 2 of the normalized data is shown, with color indicating a higher (red) or lower (blue) value than the reference strain. The clustering of parameters and strains shows Euclidean distance.

The relative end point concentrations of primary fermentation metabolites, as well as the kinetics parameters, are shown in Figure 2 for conditions NS:Normal (A), NS:Low (B) and S:Normal (C). All the strains, including C9, finished fermentation, with sugar values below 2 g/l (Supplementary Table S2). Overall, C9 behaved like regular wine yeast in reference to the laboratory strain, which would be white in all the cited parameters. EC1118 and DV10 were always grouped as they are genetically related, while C9 came closer to M2 under all the conditions. CSM is the strain that displayed a more distinctive phenotype for all the conditions. Fixing enological parameters did not change strain arrangement (data not shown). Lag phase (lp) was shorter for all the wine strains/conditions, which indicates better adaptation to grape juice fermentation. Pyruvate production was closely related to the Iag phase, particularly when shaking was not used. High pyruvate production was the signature of the CSM strain for each condition. Acetic acid and glycerol are two key metabolites whose concentrations usually evolve together during fermentation (Remize et al., 1999). In this experiment, the same occurred, particularly under the non-shaking conditions. Shaking is the condition that generally has a stronger impact on enological parameters, with acetic acid and glycerol relatively lowering in the EC1118/DV10 group compared to the other strains. Overall, strain C9 proved fit to be used as a wine yeast reference strain for genetic manipulation given its conventional fermentative behavior.

### Phenotypic Survey of the Effect of Nutrient Signaling Pathways in the Wine Fermentation Context

**FIGURE 3:**
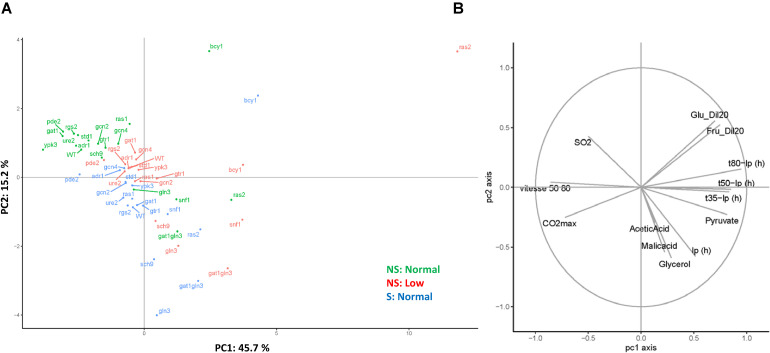
PKA mutants have a huge impact on wine fermentation. **(A)** The PCA analysis of the mutants on the nutrient signaling pathways with the three fermentations: NS:Normal (green), NS: Low (red) and S:Normal (blue). **(B)** The correlation circle of the employed kinetic and enological parameters.

**FIGURE 4:**
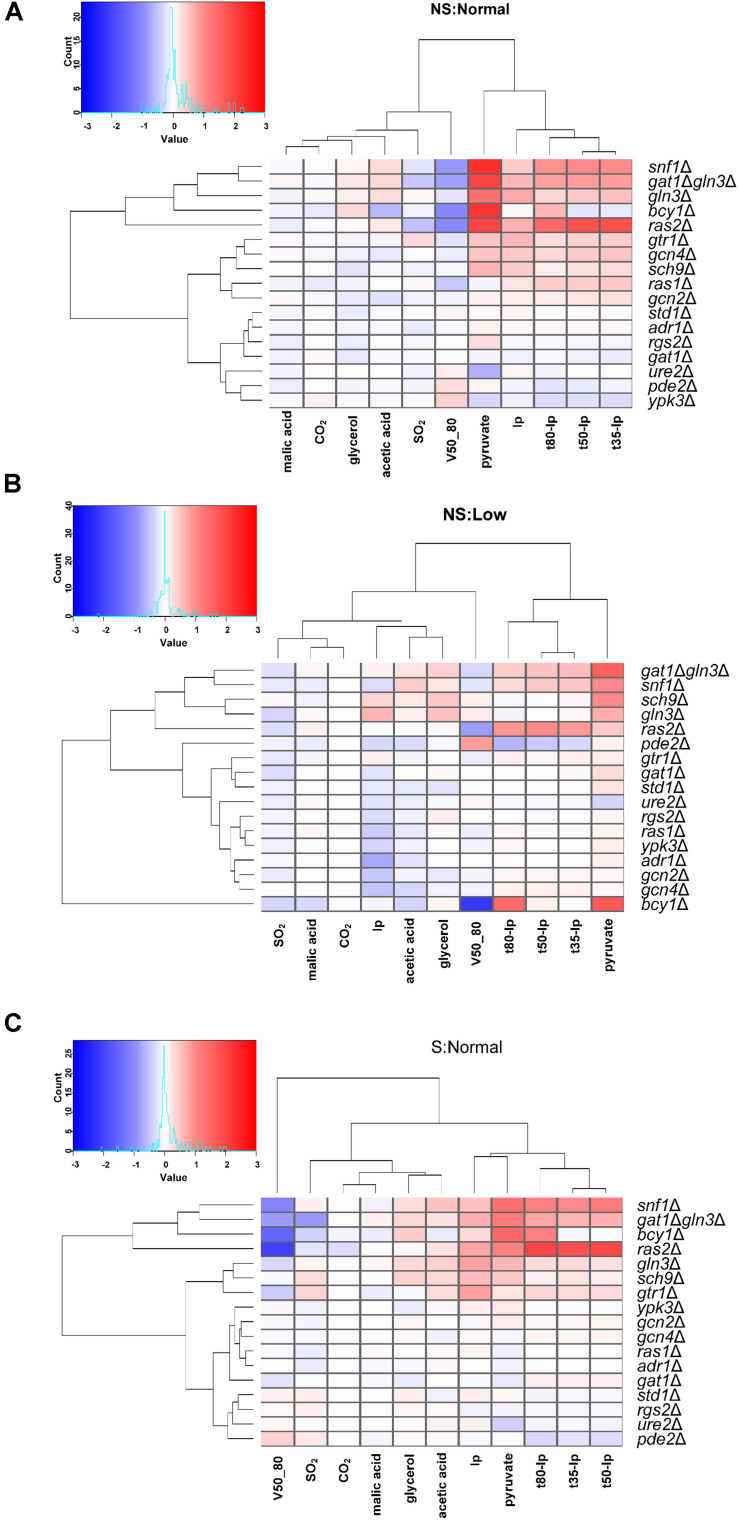
Mutants PKA, SNF1, and NCR had a huge impact on wine fermentations. Heatmaps showing relative changes in the kinetics and end-product concentrations of the selected mutants in relation to their parental strain C9. Conditions in panels **(A–C)** are explained in Figure 2.

The effect of depleting the representative genes from each signaling pathway was tested for the same three conditions (Figures 3, 4). Those include components of the PKA pathway (*RAS1/2, RGS2, BCY1, and PDE2*), TORC1 (*GTR1, SCH9, and YPK3*), GAAC (*GCN2, GCN4*), SNF1 (*SNF1, ADR1, and STD1*), and NCR (*URE2, GLN3, and GAT1*) (Supplementary Table S1 and Figure 1). Most of the null mutants completed fermentation, and rendered neither residual glucose nor residual fructose (Supplementary Table S2). However, PKA overexpression by *BCY1* deletion led to residual fructose being present, particularly for the non-shaking conditions with plenty of nitrogen, where it reached 14.4 g/l (Supplementary Figure S1). Glucose was always under 5 g/l. The *ras2* mutant only failed to fully consume fructose (19.9 g/l) and glucose (4.9 g/l) when nitrogen was limiting.

A principal component analysis (PCA) (60.9% of total variance for axes 1 and 2) was carried out to explore this multivariate dataset (Figure 3A). For each fermentation condition, most of the null mutants’ projections were localized close to the wild-type strain. However, three mutants, *ras2*Δ, *bcy1*Δ and *gat1*Δ *gln3*, were clearly discriminated by the first component (45.7% of the variance) under all the conditions. In addition, mutants *gln3D* and *sch9*Δ showed a shift in relation to the control under conditions S:Normal and NS:Low. Finally, the overall phenotype of *gtr1*Δ was impacted only under the low nitrogen conditions. This result suggests that the mutants related to the PKA pathway (*ras2*Δ, *bcy1*Δ) were impacted no matter what the condition, while the mutants on TORC1 pathway (*gtr1D* and *sch9*Δ) were more sensitive to nitrogen shortage. The second axis broadly discriminated fermentation shaking. The correlation circle (Figure 3B) indicates the correlation of kinetic parameters (t35, t50, t80) and pyruvate concentration, and their contribution to the first component.

The relative changes in the kinetics and end-product concentrations in relation to the wild-type C9 strain are shown on a heatmap for each condition (Figure 4) and all together (Supplementary Figure S2). For enological practices, the NS:Normal condition (without shaking and 250 mg/L N) could be considered standard. The other two conditions (NS:Low and S:Normal) represented nitrogen starvation and oxygen input according to the extreme situations noted for enology (Peltier et al., 2018). Under standard conditions (Figure 4A), five mutants (*bcy1*Δ, *ras2*Δ, *snf1*Δ, *gln3*Δ, *gln3*Δ *gat1*Δ) drastically affected most of the kinetic parameters, as well as pyruvate production. For these strains, on average fermentations were slower and pyruvate production drastically increased from 2-fold to almost 5-fold (Table S2). These deletion mutants included cAMP-related genes *bcy1*Δ, *ras2*Δ *and snf1*Δ, as well as NCR GATA transcription factors *gln3*Δ and *gln3*Δ*gat1*Δ. The phenotypic profile of double mutant *gln3*Δ*gat1*Δ was similar to *gln3*Δ, which is consistent with the fact that the *gat1*Δ mutant had a very mild effect on the overall phenotype. This result suggests that *GLN3* and *GAT1* do not play the same role during wine fermentation. As expected, *ure2*Δ had opposite effect to *gln3* D, especially with pyruvate production (0.63-fold vs. 2.62-fold) and to a lesser extent on the fermentation kinetics (1.1-fold vs. 0.85-fold). Likewise, the role of protein Ras2 had a stronger impact on fermentation than Ras1 under winemaking conditions. Indeed *RAS2* deletion strongly prolonged the fermentation time and slowed down fermentation speed (0.48-fold of V50_80), which was expected for the fermenting yeast cells with less PKA activity. Surprisingly, the inactivation of Ras2, an adenylate cyclase activator, had a similar effect to that caused by the inactivation of Bcy1, an inhibitor of the PKA complex. Therefore, PKA activity also has to be finely tuned during grape juice fermentation. To a lesser extent, a second mutation cluster, including the TORC1 (*gtr1*Δ, *sch9*Δ) and GAAC pathways (*gcn2*Δ and *gcn4*Δ), slightly decreased the fermentation kinetics. At the other end, deletions *URE2*, *PDE2* and *YPK3* had the most positive effect in kinetic parameters terms. *PDE2* is a cAMP degrading enzyme whose deletion increases PKA activity, which reinforces the role of PKA during winemaking. Ypk3 is a kinase-phosphorylating ribosomal protein Rps6 and is ultimately controlled by TORC1. It seemed to play a repressive role during winemaking.

Under poor nitrogen conditions (100 μg/l of YAN), a similar trend was observed for mutants *ras2*Δ, *bcy1*Δ, *snf1*Δ and *gln3*Δ*gat1*Δ (Figure 4B). *BCY1* deletion had the most remarkable effect on fermentation speed at the end of the process, although its lag phase was not affected in relation to wild-type strain C9. Under low nitrogen conditions, the inactivation of TORC1 dependent kinase *Sch9* prolonged the lag phase and increased glycerol and pyruvate production. A similar phenotypic profile was observed for the deletion of *GLN3*, which also showed a faster final speed that indicates a role in adaptation to nitrogen scarcity. *PDE2* deletion obtained the fastest fermentation speed. Regarding the chemical parameters, once again pyruvate was linked with fermentation times and, in this case, Ip did not cluster with fermentation speed, which reinforces the relevance of adaptation under these poor nitrogen conditions.

Figure 4C corresponds to the fermentation with plenty of nitrogen, but with shaking. Once again, mutants *snf1*Δ and *gln3*Δ *gat1*Δ clustered together in close proximity to deletions *BCY1* and *RAS2*. The members of TORC1 pathways SCH9 and *GTR1* shared similar profiles. GAAC members *GCN2* and *GCN4* seemed less relevant under these conditions, as did the *YPK3* kinase mutant. Adr1 is involved in the activation of respiratory genes, like *ADH2* (Beier et al., 1985). Its deletion had no major impact, which does not suggest a pressing need for respiration. However, the pyruvate level did not rise as much in some mutants, which might indicate a somewhat increased mitochondrial activity. The pyruvate concentration once again clustered with fermentation time, and also with lp again, which did not seem to strongly depend on shaking.

Two additional mutants were tested, but under all three conditions for technical reasons (Supplementary Figure S3). The mutant in transcription factor *RGT1*, which controls the expression of hexose transporters, was tested under the conditions of plenty of nitrogen, both without and with shaking (Supplementary Figures S4A,B). For both conditions, it was clustered to the *PDE2* deletion mutant, which suggests that its activity was linked with cAMP levels and, like the *pde2*Δ mutant, it increased fermentation speed. Hence hexose transport does not seem to be a limiting factor during wine fermentation. The *TOR1* kinase mutant was tested only under the low nitrogen conditions (Supplementary Figure S3C). Its impact was similar, but to a lesser extent, to that of the deletion in *GTR1*, a TORC1 activator, with extended Ip and impaired speed. Tor2 can replace Tor1 in the complex, which may explain the mild impact of this mutation (Conrad et al., 2014).

### Mutants That Show an Interaction With the Environment Belong to all the Pathways

In the previous section, the overall impact of nutrient signaling pathways was observed for many enological traits. Major effects occurred in the kinetic parameters, pyruvate production and, to a lesser extent, in the glycerol, acetic acid and SO_2_ levels. Other traits, such as malic acid concentration or produced maximal CO_2_, were mostly steady. However according to the imposed environmental conditions, each trait showed variability, as illustrated in Supplementary Figure S4 for wild-type strain C9. Compared to the reference conditions (NS:Normal), shaking prolonged the lag phase and enhanced pyruvate production, while nitrogen depletion affected only that last parameter Both shaking and low nitrogen (to a greater extent) delayed the fermentation time compared to the NS:Normal fermentation by increasing t50-Ip. Acetic acid lowered under the shaking and low nitrogen conditions, and no significant change in glycerol took place between samples.

**FIGURE 5:**
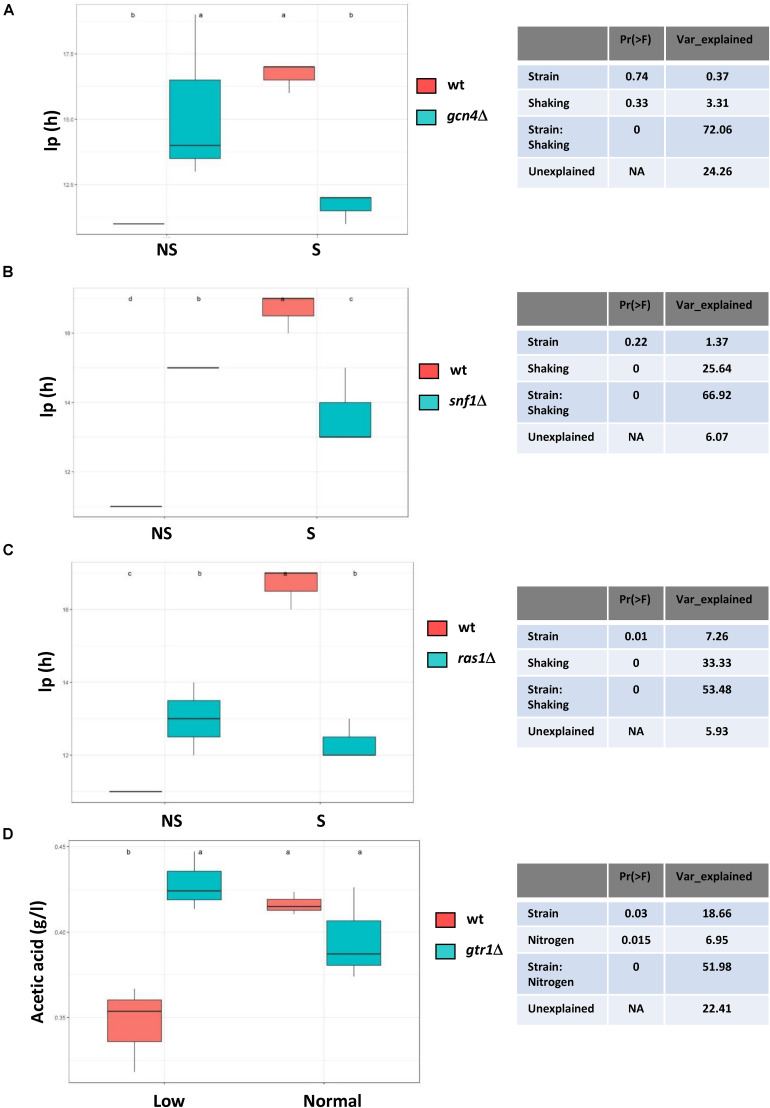
Genome-environment interaction effect on some parameters of enological interest. Four cases are shown for which variance was caused by the mutation: the environment interaction was over 50%. **(A)** lag phase time without and with shaking in mutant *gcn4*Δ. **(B)** lag phase time without and with shaking in mutant *snf1*Δ. **(C)** lag phase time without and with shaking in mutant *ras1*Δ. **(D)** Acetic acid production with low and normal nitrogen in mutant *gtr1*Δ. The shown values are the means of three replicates. Error bars represent standard error. Statistically different parameters are indicated by a different letter.

Besides the study on the environmental effect, the applied multi-environment design allowed us to assess the genotype per environment interaction (GxE). For each deletion mutant in this study, an estimation of the effect of mutation (G), environment (E), and their possible interaction (GxE), was made for each parameter by applying a two-way analysis of variance. In four deletions, the GxE effect explained more than 50% of total phenotypic variance (Figure 5). The deletion of *GCN4*, *SNF1* and *RAS1* negatively impacted Ip in the unshaken fermentations. However, these mutations shortened the lag phase when shaking was applied, thus their function was deleterious for adaptation with greater oxygen availability. Regarding nitrogen, the *gtr1*Δ mutant produced more acetic acid when the nitrogen concentration was low, but a similar amount when nitrogen was plentiful (Figure 5D). There were many other cases like this one in which the mutation had an effect under one condition, but not under another (although the percentage of variance was lower than 50%, the GxE interaction still explained part of it). Supplementary Figure S5 describes some of the most interesting cases. For instance, Ip in mutants *adr1*Δ and *gcn2*Δ remained unchanged without shaking, but reduced when shaking was applied and, alternatively, the mutations in *RAS2* and *GTR1* had prolonged lag phases for the NS:Normal condition, similarly to the S:Normal fermentation (Supplementary Figure S5A). In the low nitrogen condition, mutant *sch9*Δ produced more glycerol and acetic acid, as previously seen in synthetic must (Vallejo et al., 2017), but that difference disappeared when high nitrogen was present (Supplementary Figure S5B). The mutations in *BCY1* and *YPK3* delayed the start of fermentation by increasing Ip under the low nitrogen conditions. Interestingly, *gcn4*Δ extended Ip when nitrogen was high, but there was no difference for the low nitrogen conditions.

### The Ras System Plays a Relevant Role in Wine Fermentation and Glucose Repression

**FIGURE 6:**
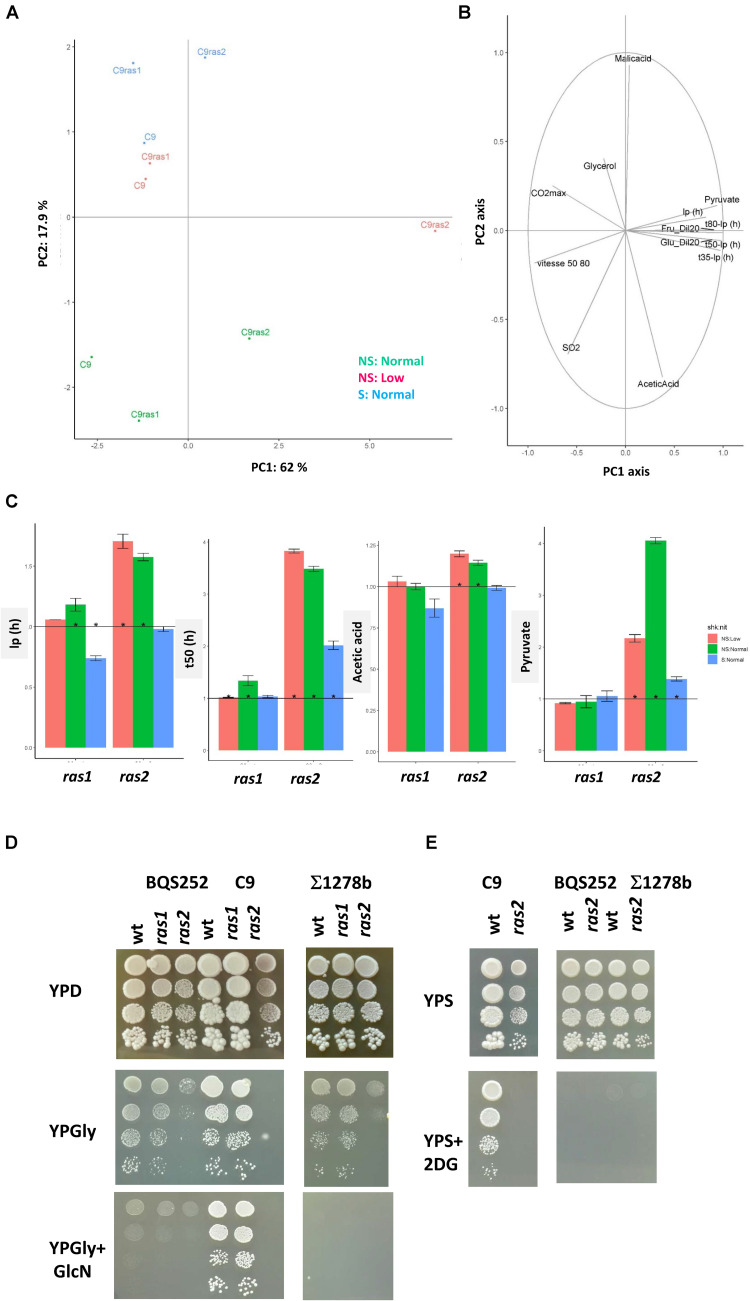
Ras2 had a profound impact on wine fermentation, respiration and glucose repression. **(A)** The PCA analysis of mutants *ras1*Δ and *ras2*Δ and parental strain C9 in the three fermentations: NS:Normal (green), NS: Low (red) and S:Normal (blue). **(B)** The correlation circle of the used kinetic and enological parameters. **(C)** Diagrams showing the relative values of lp, t50-lp (h), acetic acid, and pyruvate of mutants *ras1*Δ and *ras2*Δ in relation to parental strain C9. Fermentations were carried out in triplicate, and average and standard deviations are shown. Significant differences (*p* < 0.05) are marked by ^∗^. **(D)** The spot analysis of mutants *ras1*Δ and *ras2*Δ in three genetic backgrounds, BQS252, C9, and Σ1278b in rich medium YPD, respiratory medium YPGlycerol (YPGly) and YPGlycerol with 0.05% glucosamine (GlcN), to test glucose repression. **(E)** The spot analysis of mutant *ras2*Δ in the same genetic backgrounds grown in YP Sucrose (YPS) and YP sucrose + 100 μg/ml 2-deoxyglucose (2DG) to test glucose repression.

In the following sections, particular families of genes were studied in more detail. Mutants *ras1*Δ and *ras2*Δ are compared in Figure 6. The PCA analysis of the wild-type strain C9 and both mutants for all three conditions is shown in Figure 6A. The first component explained 62% of variance, and mutant *ras2*Δ was the main cause of this variance. This was particularly true for the NS:low condition, as mentioned above. The second component separated reference condition NS:Normal from the other two conditions, and mutant *ras1*Δ was always in close proximity to the reference strain. The correlation circle (Figure 6B) showed that fermentation times, lp and pyruvate production were behind most variance. Panel 5C shows some relative parameters of interest compared to the wild-type strain. The lag phase increased under two conditions, but not for S:Normal in mutant *ras2*Δ, where it did not change. In mutant *ras1*Δ, Ip reduced under S:Normal. Besides, the time it took to produce 50% CO_2_ always increased for this mutant, while mutant *ras1*Δ only caused a slight delay under the NS:Normal conditions. Similar results were obtained for t35-lp and t80-Ip and, therefore, fermentation speed slowed down in *ras2*Δ (data not shown). The *RAS2* deletion led to a higher pyruvate concentration under all the conditions, which was not matched by deletion *RAS1* in any case. Acetic acid increased in mutant *ras2*Δ when shaking was not used. Therefore, both Ras proteins are not interchangeable, and Ras2 plays a major role under winemaking conditions.

The Ras system is one of the two GTPase mechanisms that activate adenylate cyclase. The other one was based on the Gpr1 receptor. Rgs2 is a negative regulator that activates the GTPase activity of heterotrimeric G protein alpha subunit Gpa2 (Versele et al., 1999). A close inspection of the sequence of this gene indicated that the industrial yeasts had an extended Ct region compared to the reference laboratory strain (Supplementary Figure S6A). That region was conserved in the other species of the *Saccharomyce*s genus (Supplementary Figure S6B), which means that this gene is worth studying in the winemaking context. Due to its molecular function, an opposite phenotype to mutations *RAS* was expected, like shorter Ip and fermentation time. A significant reduction in Ip took place under the S:Normal condition, which also happened in mutant *ras1*Δ (Supplementary Figure S6C). t50-lp became slightly shorter, and only significantly so under NS:Low. Under these conditions, deletion *RAS2* considerably prolonged fermentation times. There was no alteration to metabolite production, so the impact of this protein on wine fermentation was very much limited.

Next additional growth tests were performed with different carbon sources and distinct inhibitors of nutrients signaling pathways to obtain a better understanding of the role played by Ras proteins in the physiology of wine yeasts. Deletions *RAS1* and *RAS2* were performed in laboratory strain BQS252, a strain of the S288c genetic background that has no prototrophy for amino acid biosynthesis, and also in Σ1278b, a strain with distinctive nutrient signaling; for instance, it has an overactive Ras-cAMP pathway and produces a higher peak in cAMP (Gonzales et al., 2013). As Ras2 has been linked with respiration (Tatchell et al., 1985), spot analyses on glycerol were performed (Figure 6D). In all the genetic backgrounds, deletion *RAS1* did not impact growth in a non-fermentative carbon source, but deletion *RAS2* markedly reduced growth in the laboratory strains, with no growth at all in the C9 wine strain background. On the glycerol plates, glucose repression was tested using a non-assimilable glucose analog like glucosamine. The laboratory strains displayed tight glucose repression and growth was greatly impaired as seen before for commercial yeasts (Vallejo et al., submitted). Wine strain C9 was not affected under these conditions, nor was the mutant in *RAS1*. The *RAS2* deletion mutant still did not grow. To test the *RAS2* function, another glucose-repressible carbon source was used, e.g., sucrose (Figure 6E). Under this condition, *ras2*Δ grew because the sucrose hydrolysis products could be fermented. 2-deoxyglucose, a potent glucose analog, completely prevented the laboratory strains using sucrose. C9 was more tolerant, but deletion *RAS2* completely prevented growth (deletion *RAS1* had no effect; data not shown). Therefore, Ras2 is a key factor for glucose repression regulation control in wine strains.

### Deletion of *PDE2* Improves Fermentative Performance

**FIGURE 7:**
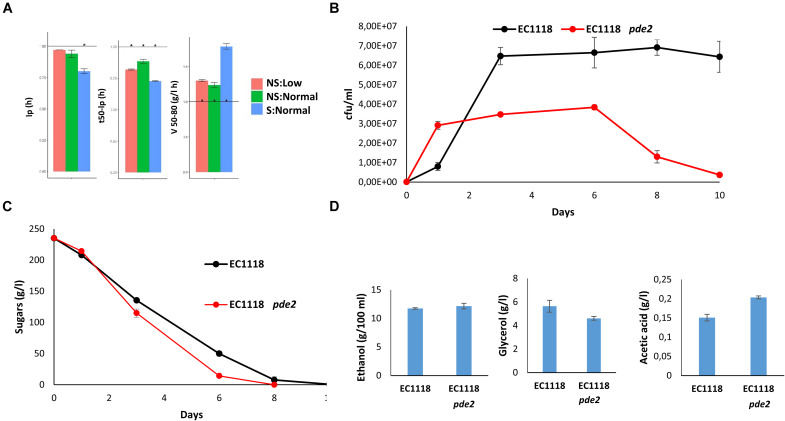
cAMP phosphodiesterase *PDE2* deletion increases fermentation speed. **(A)** Normalized values of the lag phase (lp), t50-lp, and V50-80 of mutant *pde2*Δ in relation to parental strain C9. Fermentations were carried out in triplicate, and average and standard deviations are shown. Significant differences (*p* < 0.05) are marked by *. **(B)** Cell growth measured as CFU during the red grape juice fermentation of strains EC1118 and EC1118 *pde2*Δ. **(C)** Reducing the sugar consumption of the fermentations depicted in (panel **(B)**). **(D)** Ethanol, glycerol and acetic acid production after completing these fermentations. Experiments were performed in triplicate, and average and standard deviations are shown.

Deletion *PDE2* was the only mutation to reduce all the fermentation times, t35-lp, t50-lp and t80-Ip, and fermentation speed under all the conditions (Figure 7A and data not shown). The lag time also became significantly shorter under the S:Normal conditions. Therefore, the increase in cAMP that could follow the deletion of phosphodiesterase would improve growth and metabolism, as expected. The next step was to validate this result in a different genetic background, growth medium and by another way of measuring growth and fermentation. *PDE2* was deleted from commercial diploid strain EC1118 by CRISPR-Cas9 gene editing. Small-volume fermentations were carried out in natural red grape juice and fermentation was followed by measuring CFUs (Figure 7B) and sugar consumption (Figure 7C). The engineered strain started growing earlier, but reached a lower cell density and underwent premature loss of viability in relation to the reference strain. This matches greater PKA activity, which promotes growth, but can cause problems when cells have to enter the stationary phase. Sugar consumption was faster in the mutant, even though the EC1118 strain has good fermentative power. Both strains finished fermentation, but EC1118 *pde2*Δ did so slightly earlier. There was no significant change in either ethanol or glycerol accumulation at the end of fermentation (Figure 7D), but acetic acid in the mutant significant increased. No significant change in either acetic acid or glycerol production was observed during the fermentation with C9 *pde2*Δ (data not shown). According to the *Saccharomyces* Genome Database (SGD, yeastgenome.org), *PDE2* deletion increases the sensitivity to several stress conditions in laboratory strains. EC1118 *pde2*Δ showed no decrease tolerance toward cold, oxidative, saline and copper stresses (Supplementary Figure S7). However, it has a mild sensitivity to cycloheximide. The most obvious phenotype is an increased sensitivity to heat, as it has been showed for laboratory strains (Jones et al., 2004). Fortunately, that should not be a problem in the modern day wine industry where temperature is controlled. Therefore, Pde2 is a key factor in controlling fermentation speed during winemaking and its inactivation is a way to increase fermentative performance.

### Nitrogen-Sensitive Pathways Play an Important Role in Wine Fermentation

**FIGURE 8:**
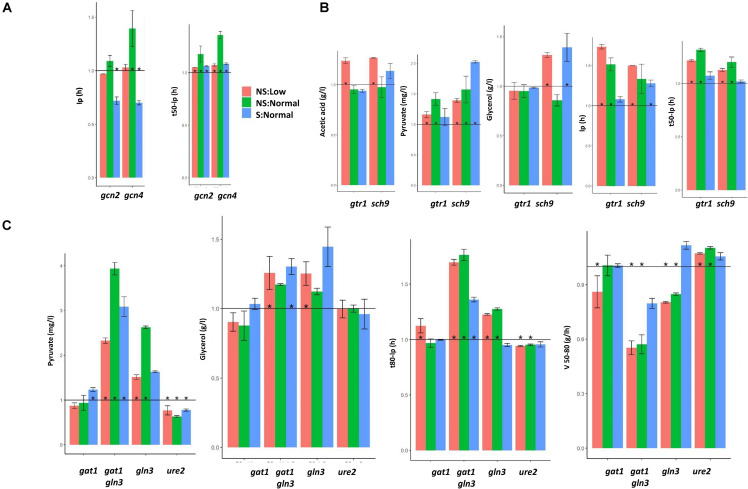
The mutations on the different nutrient signaling pathways have their own phenotypic signature. **(A)** Parameters of interest in the mutations in genes GAAC, *GCN2*, and *GCN4*. **(B)** The same for the components of TOR pathway *GTR1* and *SCH9*. **(C)** Similar analysis for NCR components *GLN3*, *GAT1*, and *URE2*. Fermentations were carried out in triplicate, and average and standard deviations are shown. Significant differences (*p* < 0.05) are marked by *.

Besides PKA, nitrogen-sensitive pathways may play a role in winemaking, such as GAAC whose main players are Gcn2 kinase and the Gcn4 transcription factor. Identifying parameters that change in both mutants may provide clues about the role of this pathway. Despite being a pathway that controls metabolism, there is no common trend in the level of relevant metabolites (data not shown), although they impact the kinetic parameters (Figure 8A). Fermentation times significantly prolonged for all the conditions, so a *de novo* amino acid biosynthesis was required later in wine fermentation. Ip in the S:Normal fermentation decreased, which indicates that GAAC plays a negative role at the very beginning of fermentation under this condition. However, Ip increased without shaking, mainly in mutant *gcn4*Δ, so GAAC plays a role in adaptation, which is sensitive to environmental conditions.

A similar approach can be followed with two factors of TORC1, *GTR1* and *SCH9* (Figure 8B). In this case, the impact on metabolite production was relevant. Both mutations increased pyruvate and acetic acid, which indicates a role in the central carbon metabolism. However, as only *sch9*Δ increased glycerol, this particular pathway had a different set of inputs. The pathway was required for adaptation as lp increased when nitrogen was low, and fermentation speed slowed down without shaking, which indicated a need for TORC1 activation at longer fermentation times.

Regarding NCR, the simplistic model indicated that Gat1 and Gln3 were partially redundant activators, and Ure2 was a repressor of both. Thus we expected the opposite results in their deletions. The deletions of *GLN3*, particularly the double *gat1*Δ *gln3*Δ mutant delay fermentation, prolonged fermentation times and slowed down V50_80 speed (Figure 8C), while *ure2*Δ had the opposite effect without shaking. The same applied for pyruvate production, which increased in deletion *GLN3* and decreased in deletion *URE2*, which suggests that fermentative metabolism is regulated by nitrogen-sensitive transcription factors during winemaking. *gln3*Δ overaccumulated glycerol under some conditions, but *ure2*Δ did not, which means that some pathways are controlled by Gln3 in a Ure2-independent way.

### Gln3 Plays an Unexpected Role in Respiration and Glucose Repression

**FIGURE 9:**
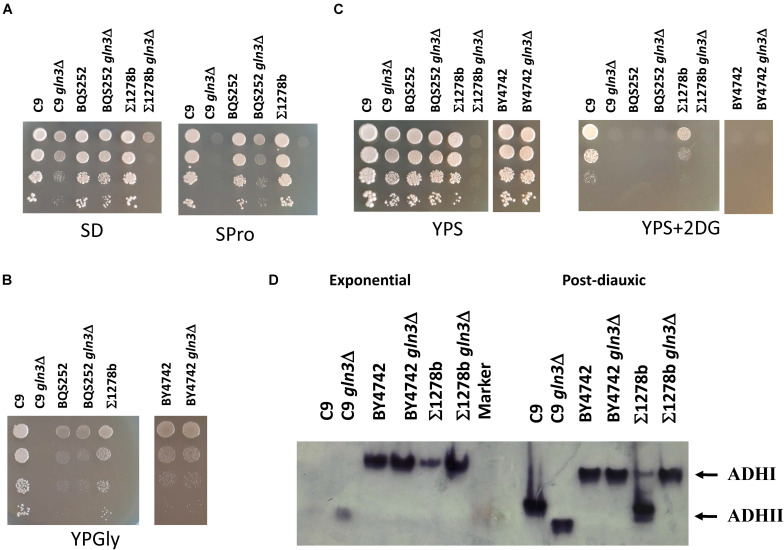
Gln3 is required for respiration and glucose repression. **(A)** The spot analysis of mutants *gln3*Δ in three genetic backgrounds, BQS252, C9, and Σ1278b in medium with ammonium as the nitrogen source (SD) and proline as the nitrogen source (SPro). **(B)** The spot analysis of the same mutants in YPSucrose with or without 100 mg/ml of 2-deoxyglucose (2DG) to test glucose repression. **(C)** The same strains in YPGlycerol to check for respiration. **(D)** The zymogram of alcohol dehydrogenase activity. Samples of the *gln3*Δ mutants in BY4742, C9, and Σ1278b genetic backgrounds were taken under the exponential (repressing) and postdiauxic (derepressing) conditions, and run in a non-denaturing PAGE gel. Alcohol dehydrogenase isoforms I and II of strain Σ1278b are indicated by arrows.

Given the relevance of *GLN3* in fermentation, the behavior of the mutant was tested on several growth media (Figures 9A,B). Ammonium, generally regarded as a rich nitrogen source, was compared to proline, a poor nitrogen source that is often used to relieve NCR (Figure 9A). Deletion *GLN3* was tested in wine strain C9 and laboratory strains BQS252 and Σ1278b. These strains are prototrophic for amino acids, so single nitrogen sources can be tested without interferences. All the strains can grow with ammonium as the sole nitrogen source. BQS252 *gln3*Δ grew well, but C9 *gln3*Δ displayed a slight growth defect compared to its parental strain. This defect was more acute in Σ1278b *gln3*Δ. This mutant displayed a growth defect even in YPD (data not shown), thus the penetration of deletion *gln3* in this genetic background was high. Growth in proline was severely impaired in the wine strain and Σ1278b backgrounds, and was mildly impaired in the BQS252 background. This scenario indicates that mechanisms to metabolize proline are more dependent on Gln3 in the wine strain. In any case, and as expected, *GLN3* was required for full growth in the poor nitrogen source.

Next mutants were grown in a respiratory carbon source (e.g., glycerol) to test their respiratory ability (Figure 9B). As a rich medium was used, a *GLN3* deletion mutant in the standard laboratory BY4742 strain (with the S288c genetic background, such as BQS252) was included as the control. Interestingly, Gln3 was required for the respiration of wine strain C9 and strain Σ1278b as the mutants showed no growth. However, the same mutation in laboratory strains BQS252 and BY4742 displayed no evident growth defect.

In order to test glucose repression, plates were used with sucrose as the sole carbon source (YPS), either with or without 2-deoxyglucose (Figure 9C). Sucrose supports the growth of mutants *GLN3* in strains BY4742, BQS252 and C9, but Σ1278b *gln3*Δ presented a severe growth defect, which suggests defective invertase induction. Glucose repression increased in C9 when *GLN3* was absent because 2DG completely inhibited mutant growth. It was difficult to notice any effect on mutant Σ1278b as the initial growth in sucrose was already severely impaired. As previously shown in the laboratory strains, growth inhibition by 2DG was complete.

This lack of growth in alternative carbon sources could reflect the impact of mutation on the transcription of one or several Gln3 regulated genes, or on the global regulation of glucose repression. To analyze this, a molecular test was run to study glucose repression in detail. A glucose repressed form of alcohol dehydrogenase exists, *ADH2*, which is activated when glucose is exhausted, while *ADH1* is the isoform used for alcoholic fermentation. Both isoforms were discriminated by the zymogram analysis when studying the mutations on the nutrient signaling pathways in the Σ1278b genetic background (Dombek et al., 2004). Mutants *GLN3* in the C9, BY4742, and Σ1278b backgrounds were grown in rich medium YPD. Samples were taken during exponential growth (repressing conditions), and 24 h later when cells were in a postdiauxic phase, which matched the de-repressing conditions. Cell extracts were run in a native polyacrylamide electrophoresis and ADH activity was visualized in-gel (Figure 9D). In the exponential phase, only ADHI was expected. There was a single common band to the laboratory strains, but the wine isoform ran differently. Deletion *GLN3* increased the ADHI signal, and to a different extent depending on the background. Under the postanoxic conditions, the pattern of ADHII expression in the Σ1278b background was the expected one. A novel band of faster mobility corresponding to ADHII appeared, while ADHI significantly reduced. However, that de-repression did not happen in Σ1278b *gln3*Δ, which matched its reduced respiration growth. Therefore, Gln3 controlled the glucose repression mechanisms in this background. No shift took place in the BY4742 strains, and the pattern between the wild type and the mutant as the same. No works in the bibliography about zymogram analyses in this background were found, so we assumed that both isoforms could not be discriminated by this assay, but *ADH2* should be active under derepressing conditions as those strains grew well in glycerol. Under the postdiauxic conditions, the wild-type wine strain C9 produced a band that ran like the ADHII of the Σ1278b strain. That band was again absent in the C9 *gln3*Δ strain, which contained a band with faster mobility, as seen under the repressed conditions. So the defect in glucose repression was conserved in wine yeasts and confirmed the role of Gln3 in controlling catabolite repression.

## Discussion

This study aimed to gain a better understanding of the role of nutrient signaling pathways in *S. cerevisiae* during wine fermentation to be used to improve yeast performance during its biotechnological use. The approach is based on a phenomics comparative analysis of many deletion mutants under three different growth conditions using a standardized fermentation set-up. The mutations covering the main pathways were tested, which confirmed some previous knowledge about the stimuli that trigger such pathways, but also provided unexpected information about them. The conditions present in the grape juice fermentation were those typical of a batch culture, where the limiting nutrients can cause growth arrest. In this case, nitrogen is usually considered the limiting nutrient as growth stops when sugars are still abundant. That view is doubtlessly too simplistic. The ratio between nutrients sometimes proved a more marked determinant for cell viability during wine fermentation (Tesniere et al., 2013; Picazo et al., 2015) than the absolute amount of nutrients. Therefore, the interactions between signaling pathways could be a determinant to understand cellular behavior during fermentation.

It is clear that among the tested mutants, those belonging to the PKA pathways displayed a more dramatic impact on fermentation. The *BCY1* and *RAS2* deletion mutants showed the most distinctive phenotype (Figures 3, 4) and did not fully consume sugars, at least under one condition. This was particularly true of fructose that is consumed more slowly than glucose (Berthels et al., 2004). Bcy1 is a negative regulator of PKA, so *bcy1*Δ is meant to display high pathway activity that could benefit the use of glucose over fructose. Therefore, the glucophilic/fructophilic bias observed among different wine yeast species could be PKA-regulated. Marked PKA activity spells problems to enter the stationary phase, which promotes early death (Cannon et al., 1990). This proved interesting in the sparkling wine production process when cell lysis is important for wine flavor (Tabera et al., 2006). Therefore, lack of sugar consumption may indicate that PKA activity is important for sugar fermentation in the stationary phase, and is relevant because a great deal of sugar metabolism during winemaking takes place without cell division. The case of *RAS2* was less obvious. Ras2 stimulated PKA, so its mutation should cause diminished PKA activity, whose effects should be the opposite to deletion *BCY1*. However, the effects of both mutations usually went in the same direction, except for acetic acid accumulation, where opposite impacts occurred (Figure 4). The phenotype associated with lack of Ras proteins was probably more complex, with functions beyond adenylate cyclase activation. Our data clearly revealed that Ras1 and Ras2 perform no overlapping function (Figure 6). The effect of deletion *RAS2* was acute when nitrogen was low, which indicates the relevance of additional signaling that does not come when glucose is present. It is known that strains with marked RAS/cAMP pathway activity show indicative phenotypes of altered nitrogen metabolism, including sensitivity to starvation and diminished vacuolar amino acid accumulation (Markwardt et al., 1995). *RAS* influences amino acid transport, but *RAS2* deletion should increase *GAP1* transporter expression (Garrett, 2008), and not impair fermentation when nitrogen is scarce. Therefore, the physiological state during winemaking differs in the involvement of the *RAS* system to well-defined laboratory media.

A fine example of the relevance of the cAMP/PKA pathway during fermentation is the effect of high-affinity cAMP phosphodiesterase *PDE2* deletion. In theory, high cAMP levels would lead to fast growth and sugar metabolism, which was observed in both the phenomics assay and the alternative vinification analysis (Figure 7). *pde*Δ was the only mutant with enhanced kinetic performance during fermentation for all the tested conditions, which makes this enzymatic activity a potential target for wine strain improvement, albeit at a cost. High PKA activity should make entry in the stationary phase difficult and impair life span, as observed in the mutant’s growth profile, with fast loss of viability (Figure 7B). This suggests that cAMP-driven metabolic stimulation might produce lower stress tolerance as trade-off.

The deletions of the key components of the main nitrogen-sensing pathway TORC1, such as *SCH9* and *GTR1*, had a strong impact when nitrogen was scarce, while the key factors in GAAC, Gcn2 and Gcn4 generally had a mild impact on fermentation when deleted. However, the deletion of the key kinase involved in glucose derepression, Snf1, also had a mayor impact for all three conditions. So in general, it would seem that carbon-responsive pathways play a more determinant role than nitrogen-responsive pathways. It is difficult to compare mutants in absolute terms. There are components of the TORC1 complex, like *KOG1*, whose deletion is lethal, as is the deletion of both genes *TOR1* and *TOR2.* This indicates the requirement of TORC1 control for growth under winemaking conditions, but basal activity probably suffices for most of the pathways’ physiological functions. Yet besides the global picture, these experiments provided a great deal of information on the genotype-environment interaction, and revealed unexpected relations. When considering the variance that may be explained by the GxE interaction, the lag phase emerged as the key parameter, which indicates that adaptation to environmental conditions took place right at the beginning of fermentation, and probably all the pathways received inputs to modulate their activity (Figure 5 and Supplementary Figure S5). More often than not, very little attention is paid to the lag phase in winemaking as it is short compared to the bulk of fermentation time. The use of mutants implied that the inoculated cells came from a stationary culture in laboratory growth media, which could differ from the conditions found in the industrially produced active dry yeasts, but information was extracted. The amino acid synthesis led by Gcn4 was required for fast adaptation when there was no shaking, but was detrimental when shaking took place. A similar phenotype was found for deletion mutants *SNF1* and *RAS1*. It would have been reasonable to predict a role for Snf1 under the micro-oxygenation conditions as it regulated the respiratory genes, but a role was not expected for Gcn4, which depends on the culture shaking status under winemaking conditions. TORC1 was key in the GxE interaction as far as producing enological relevant metabolites was concerned. Acetic acid accumulation was *GTR1*- and *SCH9*-dependent for nitrogen starvation (Figure 5 and S5), and glycerol production was modulated by Sch9 under these conditions. Therefore, TORC1 is the most promising target pathway to be modulated for the controlled production of wine organoleptic metabolites.

Nitrogen availability and its impact on nutrient sensing pathways, such as TORC1, is well-known and some information is emerging from winemaking conditions. The impact of oxygen on fermentation is less known, but it has been described that shaking, in this experimental setup, increases micro-oxygenation (Peltier et al., 2018). The mutations of *GCN4*, *SNF1, RAS1, ADR1* and *GCN2* reduced lp with shakings. When Snf1 was active, it produced Adr1 activation to promote respiration by inducing glucose repressed genes like *ADH2*. Alcohol dehydrogenase 2 activity seemed detrimental at the very beginning of vinification, so it would be better to keep respiratory metabolism inactivate in the early grape juice fermentation stages. The spot analysis revealed that a close link between respiratory metabolism and glucose repression. It is known that *RAS2* is required for respiratory metabolism (Tatchell et al., 1985), which was also the case in wine yeasts (Figure 6), but not for *RAS1*. Wine strains displayed constitutive high tolerance to glucose analog 2DG, which indicates low glucose repression. However, this tolerance to 2DG disappeared in the *RAS2* mutant, so whatever signaling actually caused growth in the presence of 2DG was channeled through Ras2. Lack of respiration ability in the *gln3*Δ mutant in the wine and Σ1278b genetic backgrounds was more striking because that phenotype has not been described before. Gln3 would be necessary to transcribe some gene(s) involved in respiration, but the zymogram analysis of glucose-repressive ADHII indicated that this enzyme’s mechanism of activation was blocked in these backgrounds, but not in the reference S288c backgrounds. This finding suggests a global impact on glucose repression. The *GLN3* deletion mutant had a similar fermentation profile to *snf1*Δ, lacking the AMPK enzyme (Figure 4), which reinforces the direct link of Gln3 with glucose repression. As Gln3 proved to be phosphorylated by Snf1, it would seem that agreat deal of Snf1 signaling was channeled through Gln3 during fermentation, which could impact glucose repression by direct or indirect means in some genetic backgrounds, including those of wine strains. Genetic background is a factor that has to be considered when studying nutrient signaling pathways. Strain Σ1278b shows some differences to the laboratory strains from the S288c background. For instance, *BMH1* and *BMH2* deletion is lethal in most laboratory strain genetic backgrounds (Gelperin et al., 1995), but not in this one. This could influence the glucose repression behavior of strain Σ1278b (Dombek et al., 2004) in a way that may be shared by wine strains, although sensitivity to 2DG is quite different (Figure 6E). Strain Σ1278b is thought to possess marked constitutive PKA activity (Stanhill et al., 1999), which might be the case of wine yeasts, and such marked PKA activity may be relevant for performing grape juice fermentation as the mutations in the pathway strongly influence the process. The genetic landscape is probably not homogenous among different industrial isolates that perform with their own particular imprint (Figure 2). Therefore, no nutrient signaling mechanism should be taken for granted to explain complex phenotypes, and a comparative analysis between different genetic backgrounds must carried out when studying these molecular pathways, and when they are genetically edited to boost yeast biotechnological performance.

## Data Availability Statement

All datasets presented in this study are included in the article/Supplementary Material.

## Author Contributions

BV, EP, and VG carried out the experiments. BV, EP, EM, PM, and AA analyzed the data and reviewed the manuscript. EM, PM, and AA contributed reagents/tools. AA wrote the manuscript. All authors contributed to the article and approved the submitted version.

## Conflict of Interest

EP and PM are working for the company BIOLAFFORT. This does not alter the authors’ adherence to all the journal policies on sharing data and materials. The remaining authors declare that the research was conducted in the absence of any commercial or financial relationships that could be construed as a potential conflict of interest.
