# Supplementary material for: Role of Saccharomyces cerevisiae Nutrient Signaling Pathways During Winemaking: A Phenomics Approach
Source: Front Bioeng Biotechnol. 2020 Jul 22;8:853. doi: 10.3389/fbioe.2020.00853 (PMC7387434; doi:10.3389/fbioe.2020.00853)
Supplement: Supplementary file 1 [file Image_1.PDF]

A)

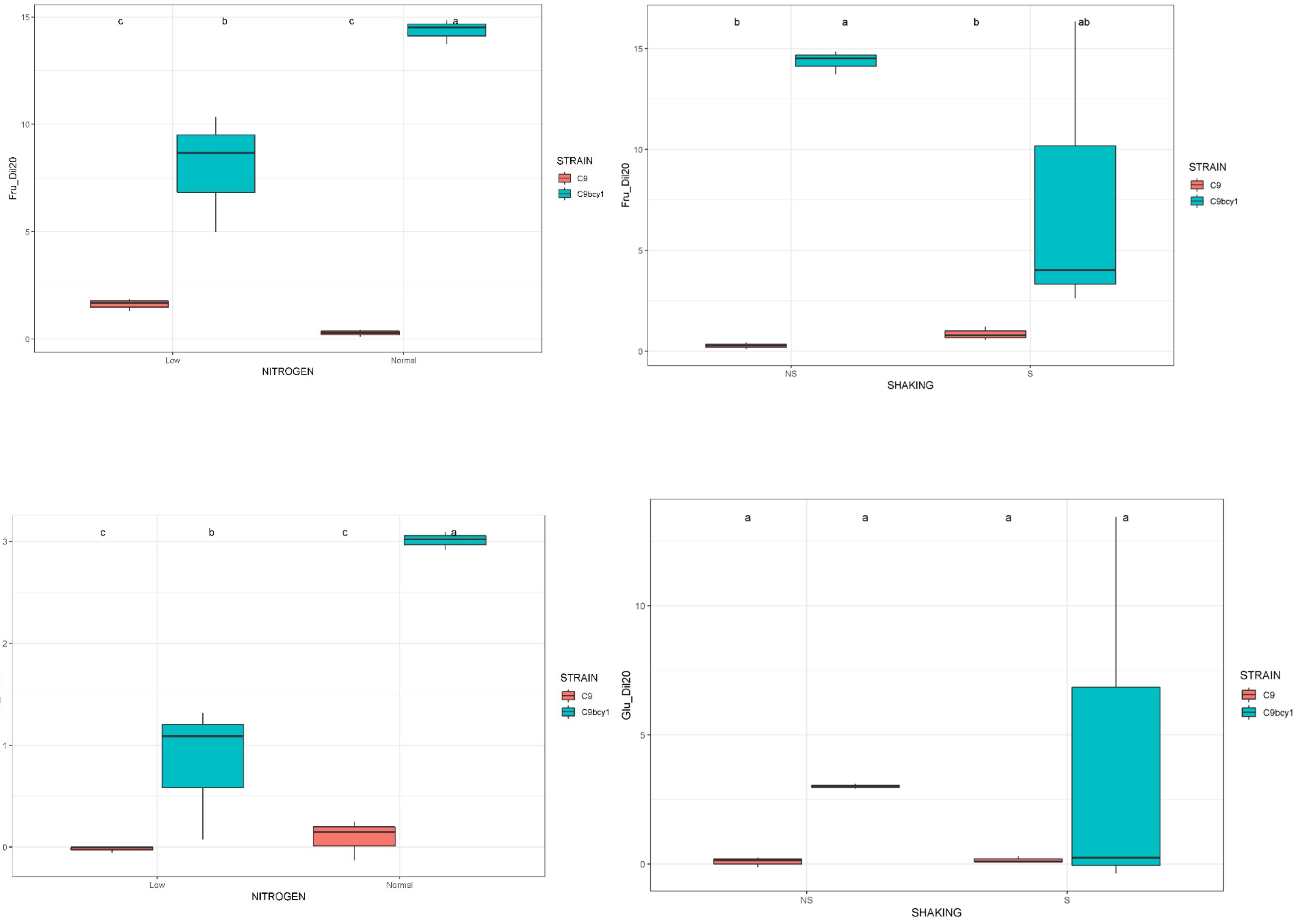

B)

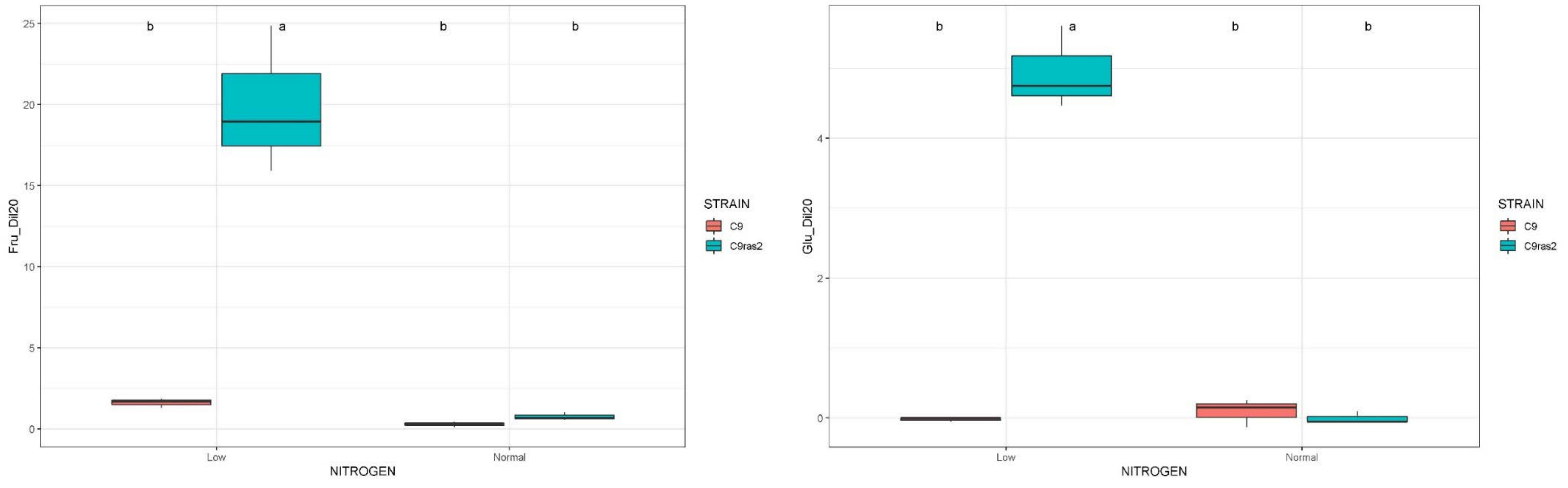

Supplemental Figure S1. A) Residual sugars of *bcy1*Δ mutant after fermentation.
B) Residual sugars of *ras2*Δ mutant at low nitrogen
