# Supplementary material for: Role of Saccharomyces cerevisiae Nutrient Signaling Pathways During Winemaking: A Phenomics Approach
Source: Front Bioeng Biotechnol. 2020 Jul 22;8:853. doi: 10.3389/fbioe.2020.00853 (PMC7387434; doi:10.3389/fbioe.2020.00853)
Supplement: Supplementary file 2 [file Image_2.PDF]

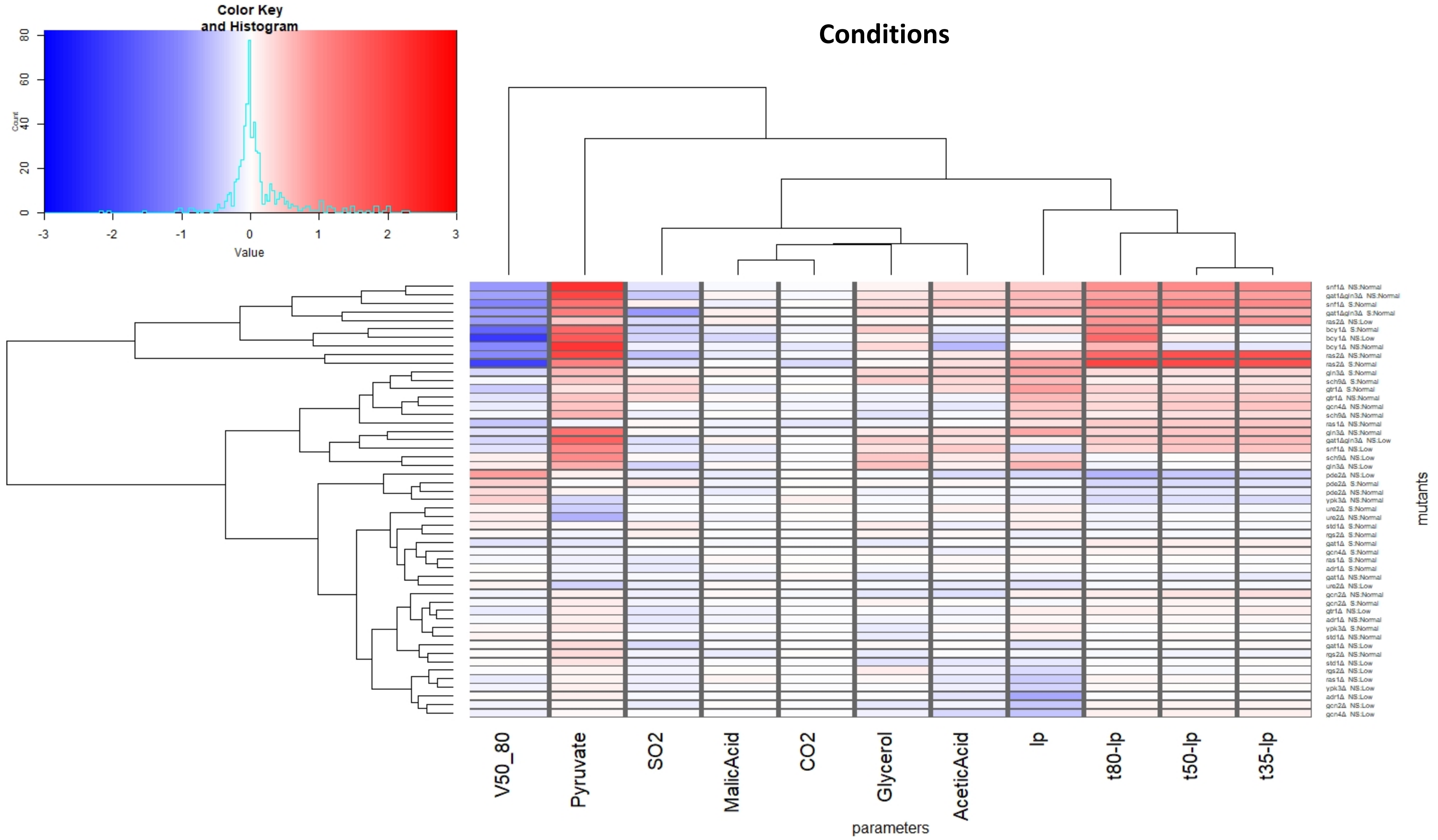

Supplementary Figure S2. Heatmaps showing relative changes in the kinetics and end-product concentrations of mutants relative to the parental strain C9 in all three conditions, Not shaking: normal nitrogen (NS:Normal), Not shaking: low nitrogen (NS:Low), and Shaking: normal nitrogen (S:Normal). Log 2 of the normalized data is shown, color indicating a higher (red) or lower (blue) value than the reference strain. Clustering of parameters and strains show Euclidean distance.
