# Supplementary material for: Role of Saccharomyces cerevisiae Nutrient Signaling Pathways During Winemaking: A Phenomics Approach
Source: Front Bioeng Biotechnol. 2020 Jul 22;8:853. doi: 10.3389/fbioe.2020.00853 (PMC7387434; doi:10.3389/fbioe.2020.00853)
Supplement: Supplementary file 3 [file Image_3.PDF]

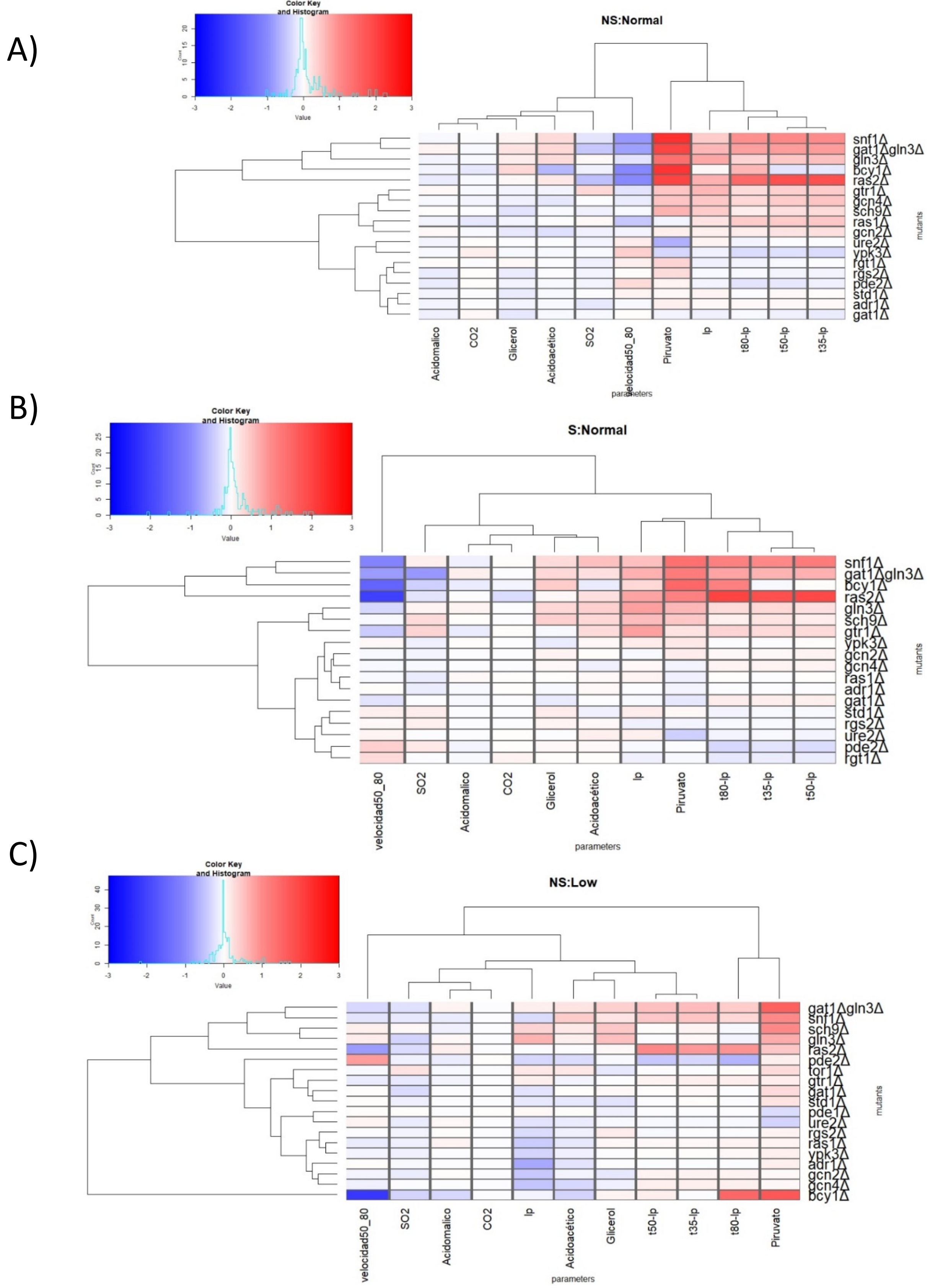

Supplemental Figure S3.Heatmaps showing relative changes in the kinetics and end-product concentrations of all mutants relative to parental strain C9. A) Not shaking: normal nitrogen, B) Not shaking: low nitrogen, and C) Shaking: normal nitrogen. Log 2 of the normalized data is shown, color indicating a higher (red) or lower (blue) value than the reference strain. Clustering of parametres and strains show Euclidean distance.
