# Supplementary material for: Role of Saccharomyces cerevisiae Nutrient Signaling Pathways During Winemaking: A Phenomics Approach
Source: Front Bioeng Biotechnol. 2020 Jul 22;8:853. doi: 10.3389/fbioe.2020.00853 (PMC7387434; doi:10.3389/fbioe.2020.00853)
Supplement: Supplementary file 4 [file Image_4.PDF]

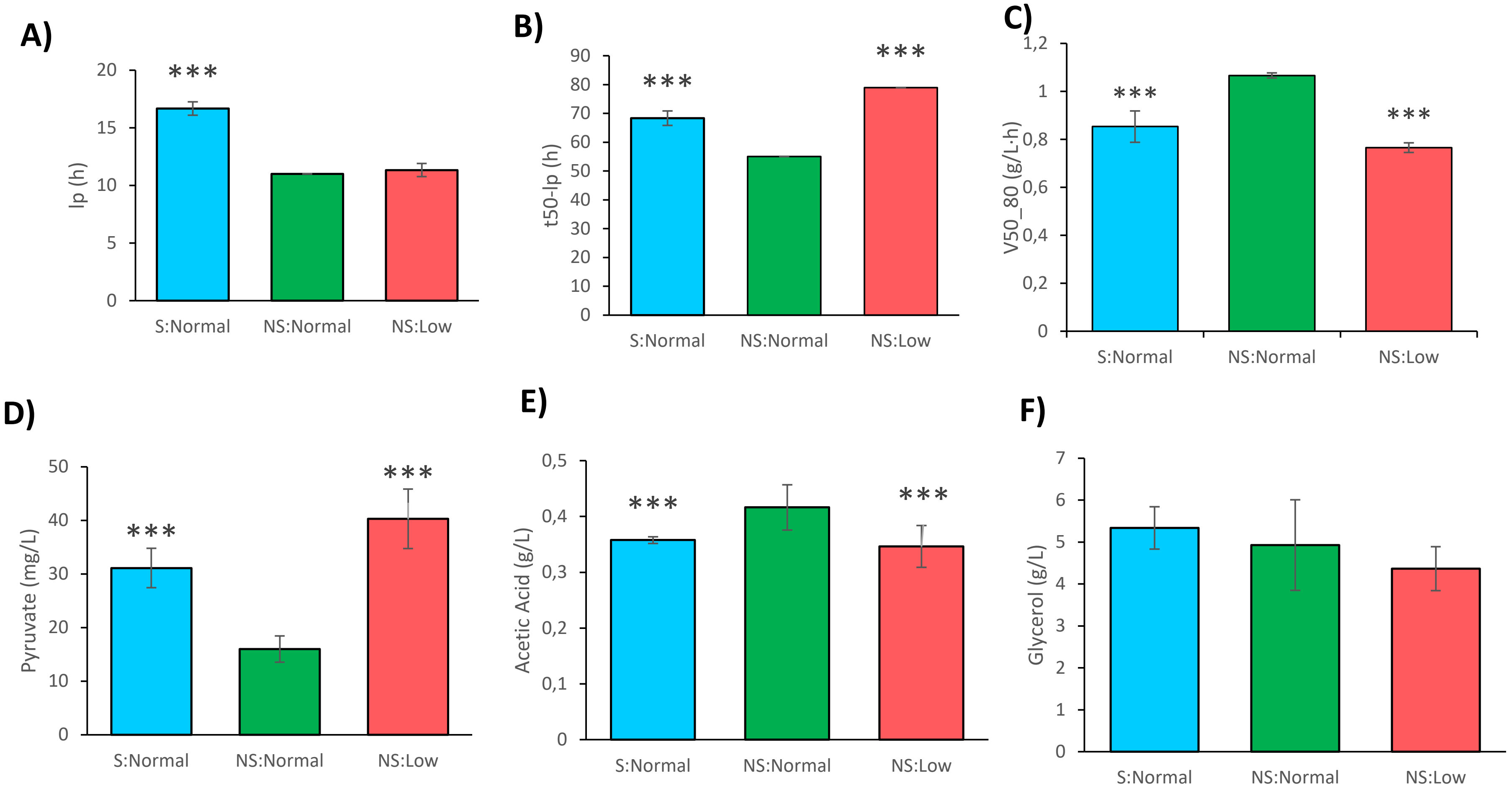

Supplementary Figure S4. Metabolite production of reference strain C9 in all three conditions: NotShaking: Normal Nitrogen. Shaking: Normal Nitrogen And Non-Shaking:Los Nitrogen. A) lag phase. B) t50-Ip. C) V50-80. D) Pyruvate. E) Acetica acid. F) Glycerol. Fermentations were carried out in triplicate, average and standar deviation is shown. Significant differences ($p<0.05$) were marked with *
