# Supplementary material for: Role of Saccharomyces cerevisiae Nutrient Signaling Pathways During Winemaking: A Phenomics Approach
Source: Front Bioeng Biotechnol. 2020 Jul 22;8:853. doi: 10.3389/fbioe.2020.00853 (PMC7387434; doi:10.3389/fbioe.2020.00853)
Supplement: Supplementary file 5 [file Image_5.PDF]

A)

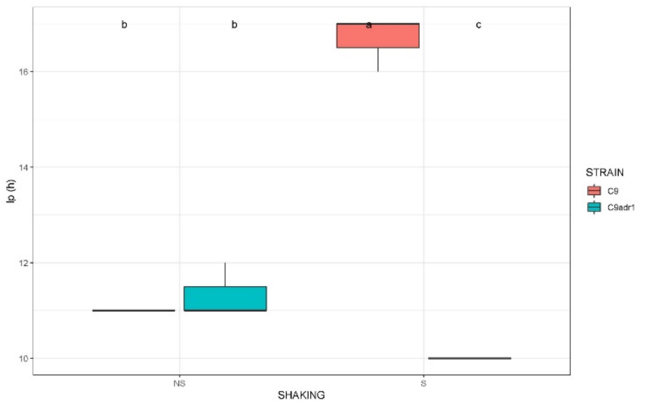

	Pr(>F)	Var_explained
Strain	0	36.58
SHAKING	0	17.12
STRAIN:SHAKING	0	44.68
Unexplained	NA	1.62

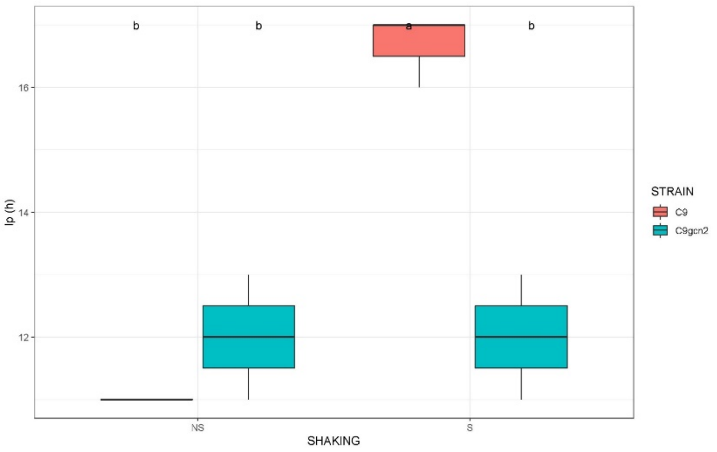

	Pr(>F)	Var_explained
Strain	0	16.03
SHAKING	0	38.28
STRAIN:SHAKING	0	38.28
Unexplained	NA	7.42

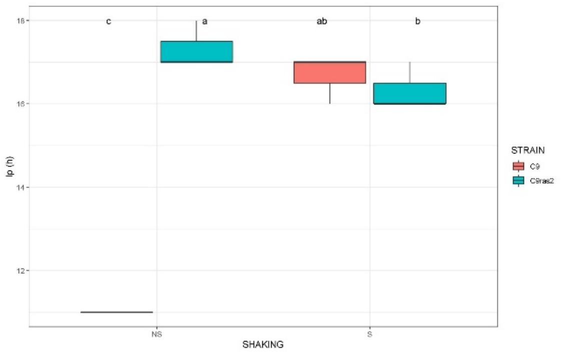

	Pr(>F)	Var_explained
Strain	0	34.32
SHAKING	0	20.76
STRAIN:SHAKING	0	42.37
Unexplained	NA	2.54

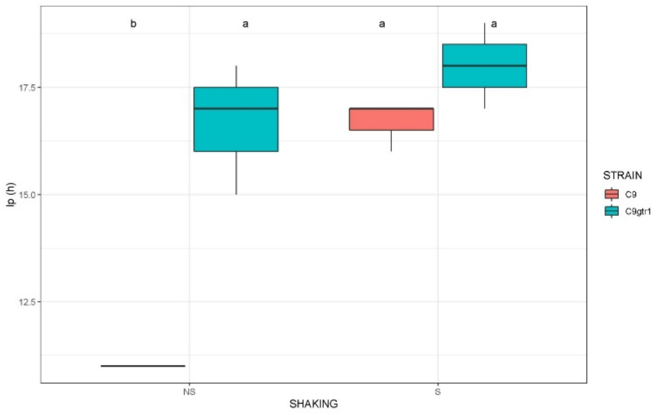

	Pr(>F)	Var_explained
Strain	0	38.72
SHAKING	0	38.72
STRAIN:SHAKING	0	14.84
Unexplained	NA	7.73

B)

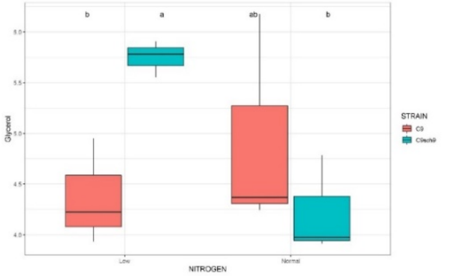

	Pr(>F)	Var_explained
Strain	0.4	4.38
NITROGEN	0.24	9
STRAIN:NITROGEN	0.02	42.22
Unexplained	NA	44.39

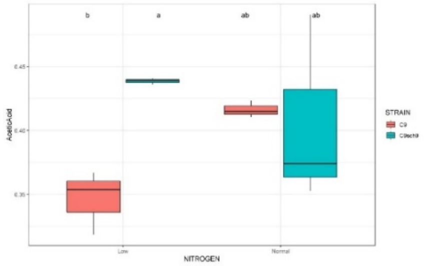

	Pr(>F)	Var_explained
Strain	0.11	18.98
NITROGEN	0.44	3.93
STRAIN:NITROGEN	0.05	30.13
Unexplained	NA	46.95

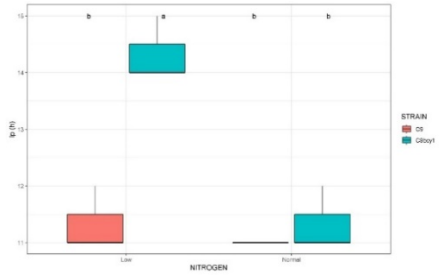

	Pr(>F)	Var_explained
Strain	0	34.72
NITROGEN	0	34.72
STRAIN:NITROGEN	0	22.22
Unexplained	NA	8.33

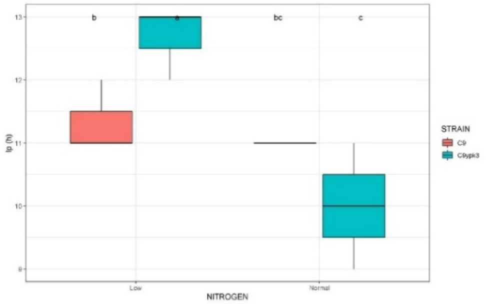

	Pr(>F)	Var_explained
Strain	0.67	0.58
NITROGEN	0	47.37
STRAIN:NITROGEN	0.01	28.65
Unexplained	NA	23.39

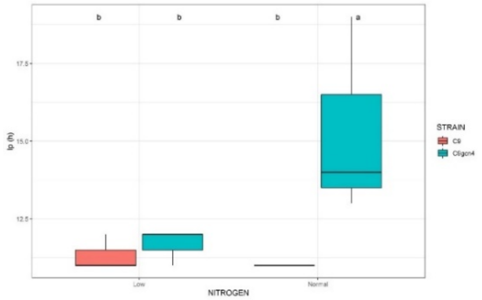

	Pr(>F)	Var_explained
Strain	0.04	27.84
NITROGEN	0.12	14.2
STRAIN:NITROGEN	0.07	20.45
Unexplained	NA	37.5

Supplementary Figure S5. Interactions gene-environment. Examples of intraction between the genotype and the environmental conditions, when the mutation has different effects in different conditions or when it is only showed in one conditions. A) Effect of shaking. B) Effect of nitrogen amount.
