# Supplementary material for: Role of Saccharomyces cerevisiae Nutrient Signaling Pathways During Winemaking: A Phenomics Approach
Source: Front Bioeng Biotechnol. 2020 Jul 22;8:853. doi: 10.3389/fbioe.2020.00853 (PMC7387434; doi:10.3389/fbioe.2020.00853)
Supplement: Supplementary file 6 [file Image_6.PDF]

A)

Rgs2 Ct

```
s288c      KGQNSHQQR*-------------------------------------------
W303       KGQNSHQQR*-------------------------------------------
Sigma      KRTKQSSTKIASSSSVETQISSSSSPLPNKAIGKNEKSVENGFKKLNLHDIS*
CLIB       KRTKQSSTKIASSSSVETQISSSSSPLPNKAIGKNEKSVENGFKKLNLHDIS*
EC1118     KRTKQSSTKIASSSSVETQISSSSSPLPNKAIGKNEKSVENGFKKLNLHDIS-
L2056      KRTKQSSTKIASSSSVETQISSSSSPLPNKAIGKNEKSVENGFKKLNLHDIS-
```

B)

SGD_Scer_RGS2/YOR107W	298	NRLKGQNSHQQR-------------------------------------	309
MIT_Smik_c592_20275	299	RHLKRAKQSSTTVSSSSSKNTESSSSPLPLINKAIDHKEKSVEKGFKKLN	348
MIT_Spar_c483_20728	298	NHLRRTKQPSTNITSSSSMKAEISLSSSPLPNKAIGQNVKSVEKGFKKLN	347
MIT_Suva_c651_22829	293	NHLKRAKQSPAITTSSS---------PSSFPPTATGLNEKSVENGFQKLN	333
**Symbols**		.:*: :.	

C)

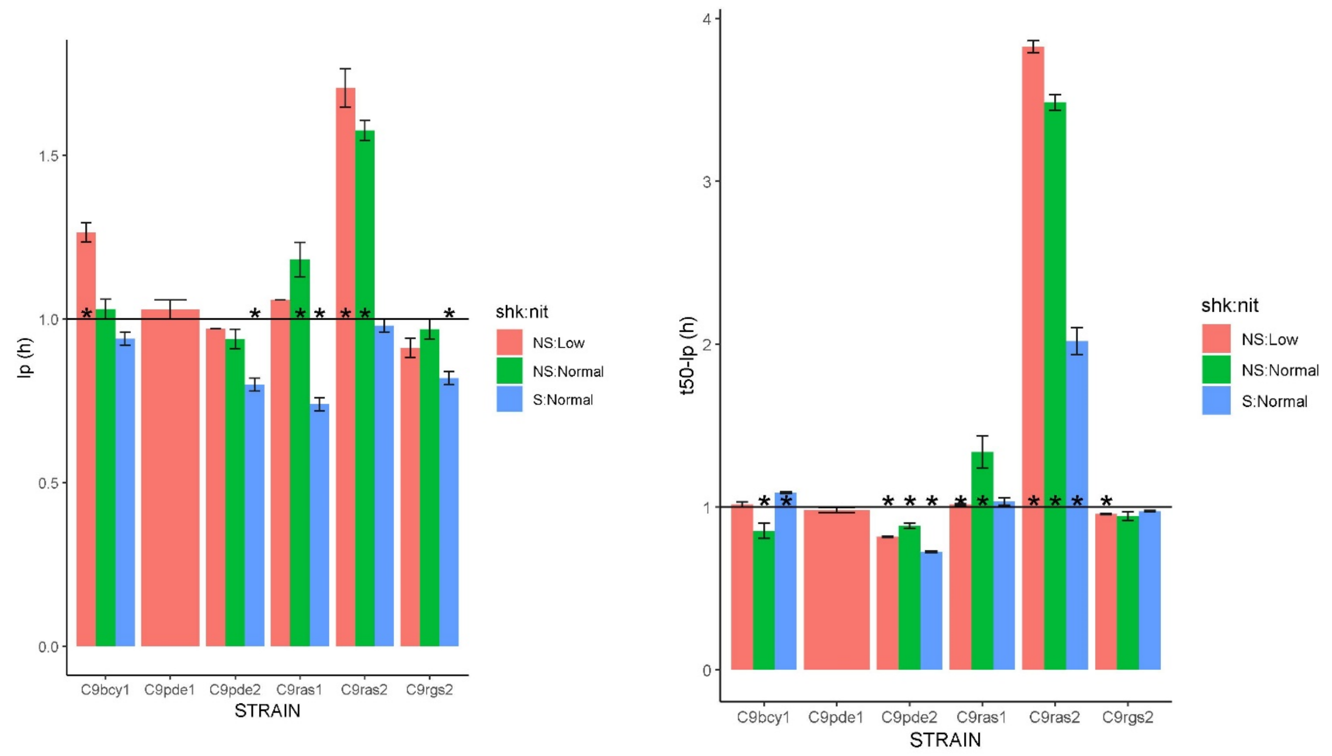

Supplemental Figure S6. A) C-terminal sequence or protein Rgs2 in several *S. cerevisiae* strains according to SGD. B) Synteny analysis of C-terminal sequence or protein Rgs2 in several *Saccharomyces* species: *S. cerevisiae, S. mikatae, S. paradoxus and S. uvarum*, according to SGD. D) lag phase and t50 of *rgs2Δ* mutant compared to its parental strain C9. Other mutants of PKA pathway are included.
