# Supplementary material for: Role of Saccharomyces cerevisiae Nutrient Signaling Pathways During Winemaking: A Phenomics Approach
Source: Front Bioeng Biotechnol. 2020 Jul 22;8:853. doi: 10.3389/fbioe.2020.00853 (PMC7387434; doi:10.3389/fbioe.2020.00853)
Supplement: Supplementary file 7 [file Image_7.PDF]

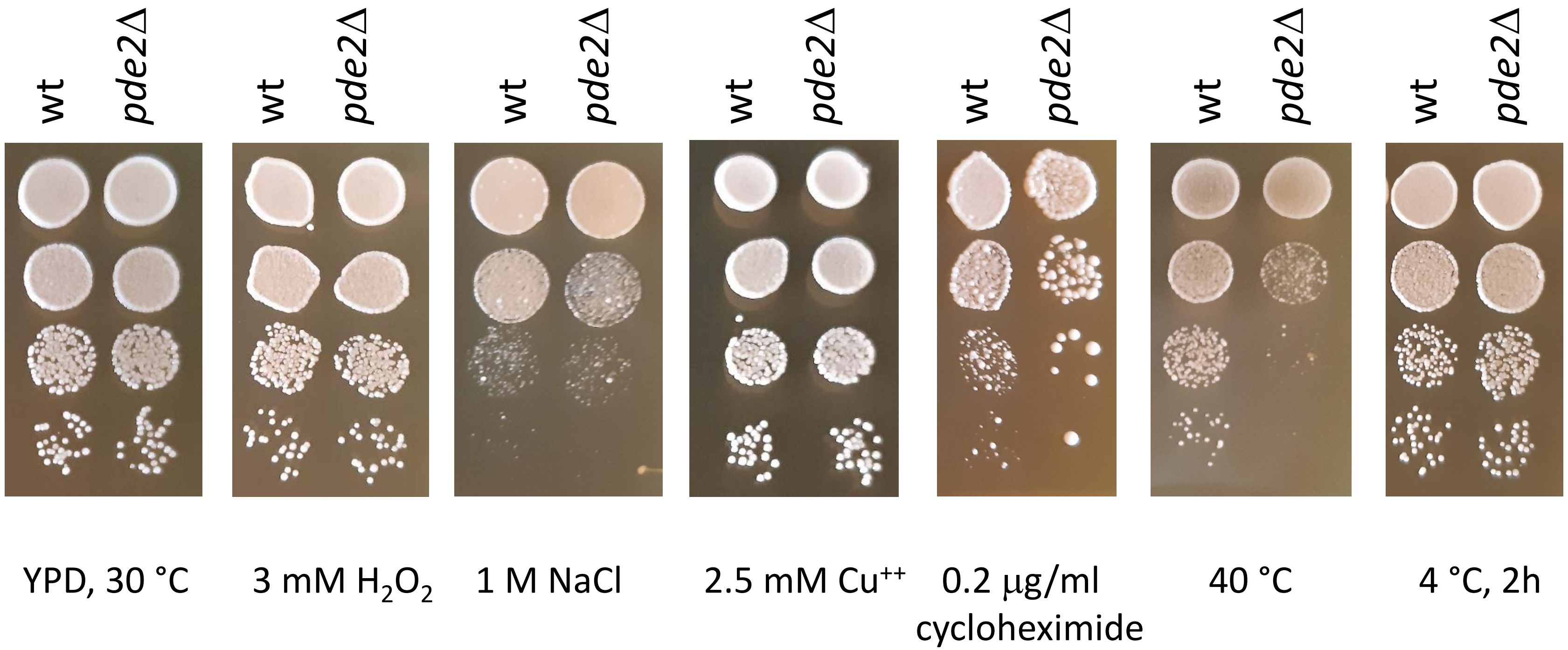

Supplementary Figure S7. Stress analysis of *PDE2* deletion. Stationary cultures in YPD of EC1118 and EC1118 *pde2Δ* strains were serially diluted and 5 µl drops were placed on YPD plates containing the indicated amount of the stress compound and incubated at 30 °C. To test high temperature the palate was incubated at 40 °C and to test low temperature the plate was incuabted at 4 °C for two hours prior to its incubation at 30 °C.
