# Supplementary material for: Role of Saccharomyces cerevisiae Nutrient Signaling Pathways During Winemaking: A Phenomics Approach
Source: Front Bioeng Biotechnol. 2020 Jul 22;8:853. doi: 10.3389/fbioe.2020.00853 (PMC7387434; doi:10.3389/fbioe.2020.00853)
Supplement: Supplementary file 8 [file Table_1.PDF]

Supplementary Table S1: strains used in this work

**Strain**	**Genotype**	**Origin**
BQS252	MAT a *ura3-52*. Spore form FY1679	J. Enrique Pérez-Ortin
BQS252 *gln3*	BQS252 *gln3::KanMX*	This work
BY4742	BY4742 MAT α his3Δ1 leu2Δ0 lys2Δ0 ura3Δ0	Brachmann et al., 1998
BY4742 *gln3*	BY4742 *gln3::KanMX*	This work
C9	C9 Mat a *ho::loxP*	Walker *et al.*, 2003
C9 *adr1*	C9 *adr1::loxP*	This work
C9 *bcy1*	C9 *bcy1::KanMX*	“ ”
C9 *gat1*	C9 *gat1::KanMX*	“ ”
C9 *gat1 gln3*	C9 *gat1::LoxP gln3::KanMX*	“ ”
C9 *gln3*	C9 *gln3::KanMX*	“ ”
C9 *gcn2*	C9 *gcn2::KanMX*	“ ”
C9 *gcn4*	C9 *gcn4::KanMX*	“ ”
C9 *gtr1*	C9 *gtr1::KanMX*	“ ”
C9 *pde1*	C9 *pde1::KanMX*	“ ”
C9 *pde2*	C9 *pde2::KanMX*	“ ”
C9 *ras1*	C9 *ras1::KanMX*	“ ”
C9 *ras2*	C9 *ras2::KanMX*	“ ”
C9 *rgs2*	C9 *rgs2::KanMX*	“ ”
C9 *rgt1*	C9 *rgt1::KanMX*	“ ”
C9 *sch9*	C9 *sch9::loxP*	“ ”
C9 *snf1*	C9 *snf1::loxP*	“ ”
C9 *std1*	C9 *std1::loxP*	“ ”
C9 *tor1*	C9 *tor1::KanMX*	“ ”
C9 *ypk3*	C9 *ypk3::KanMX*	“ ”
C9 *ure2*	C9 *ure2::KanMX*	“ ”
C9 *ypk3*	C9 *ypk3::KanMX*	“ ”
CSM	Commercial strain Enoferm CSM	Lallemand
DV10	Lalvin DV10	Lallemand
EC1118	Lalvin EC1118	Lallemand
EC1118 *pde2*	EC1118 *pde2*Δ	This work
M2	*Enoferm M2*	Lellemand
Σ1278b		G,Fink
Σ1278b *gln3*	Σ1278b *gln3::KanMX*	This work
